# Night shift work and female breast cancer: a two-stage dose-response meta-analysis for the correct risk definition

**DOI:** 10.1186/s12889-024-19518-2

**Published:** 2024-07-31

**Authors:** Jinyoung Moon, Atsuko Ikeda-Araki, Yongseok Mun

**Affiliations:** 1 Interdisciplinary Program in Bioinformatics, College of Natural Sciences, Seoul National University, 1, Gwanak-ro, Gwanak-gu, Seoul, 08826 South Korea; 2 Department of Occupational and Environmental Medicine, Inha University Hospital, 27, Inhang-ro, Jung-gu, Incheon, 22332 South Korea; 3 Center for Environmental and Health Sciences, Hokkaido University, Sapporo, Japan; 4 Faculty of Health Sciences, Hokkaido University, Sapporo, Japan; 5 Department of Ophthalmology, Hallym University Kangnam Sacred Heart Hospital, 1, Singil-ro, Yeongdeungpo-gu, Seoul, 07441 South Korea

**Keywords:** Night shift work, Breast cancer, Dose-response meta-analysis, Cohort study, Case-control study

## Abstract

**Introduction:**

The hypothesis of this study is night shift work exposure can increase the risk of female breast cancer. To validate this hypothesis, the authors conducted a two-stage dose-response meta-analysis with improved quality on this topic.

**Methods:**

The medical librarian searched PubMed, EMBASE, and the Cochrane Library on December 30th, 2022. The eight inclusion criteria were determined and strictly applied to the selection process. A reliable dose-response meta-analysis methodology was applied.

**Results:**

Reliable 10 cohort (total cases: 15,953, and total person-years: 6,812,138) and 11 case-control reports (total cases: 9196, and total controls:12,210) were included in the final analysis. The pooled risk ratio (RR) of female breast cancer (from cohort studies) for 1, 10, 20, and 30 years of night shift work exposure was 1.0042 (95% CI 1.0014–1.0070), 1.0425 (95% CI 1.0138–1.0719), 1.0867 (95% CI 1.0278–1.1490), and 1.1328 (95% CI 1.0419–1.2317), respectively. The pooled odds ratio (OR) of female breast cancer (from case-control studies) for 1, 10, 20, and 30 years of night shift work exposure was 1.0213 (95% CI 1.0108–1.0319), 1.2346 (95% CI 1.1129–1.3695), 1.5242 (95% CI 1.2386–1.8756), and 1.8817 (95% CI 1.3784–2.5687), respectively.

**Discussion:**

This study has several strengths from the perspective of a dose-response meta-analysis: Strictly applied eight inclusion criteria, separately synthesized RRs from cohort studies and ORs from case-control studies, clearly defined exposure dose, years of night shift work for each risk estimate, a reliable dose-response meta-analysis methodology, and careful considering of selection, exposure, and outcome biases and confounder adjustment for each study. This careful consideration of potential biases and confounding led to the exclusion of unreliable two cohort and five case-control studies.

**Supplementary Information:**

The online version contains supplementary material available at 10.1186/s12889-024-19518-2.

## Introduction

The relationship between night shift work and breast cancer has been discussed in many previous studies. However, a definite conclusion was not made. In a systematic review and meta-analysis study by Van et al., the pooled risk ratio (RR) from cohort and nested case-control studies were 0.98 (95% CI 0.93–1.03) and 1.14 (95% CI 0.89–1.46), respectively, without statistical significance [1]. Only the pooled odds ratio (OR) from case-control studies was 1.34 (95% CI 1.17–1.53) with statistical significance. However, this study extracted only one representative risk estimate for night shift work versus day work from each study, and these risk estimates were synthesized to calculate pooled risk estimates. In a systematic review and meta-analysis study by Manouchehri et al., the pooled RR for the subjects with < 10 years of night shift work exposure was 1.13 (95% CI 1.03–1.24) with statistical significance. However, the pooled OR for the subjects with ≥ 10 years of night shift work exposure was 1.08 (95% CI 0.99–1.17) with statistical insignificance [2]. However, this study classified the exposure dose (years of night shift work) into only two categories, < 10 years and ≥ 10 years, and did not consider a dose-response relationship.

Even though the conclusions of individual articles and meta-analyses are divergent, the biological background for the association between night shift work and breast, prostate, and colorectal cancer is rather stable. The International Agency for Research on Cancer (IARC) classified night shift work as Group 2 A (probably carcinogenic) carcinogen for these three cancers. This indicates that night shift work has limited evidence of carcinogenicity in humans, sufficient evidence of carcinogenicity in experimental animals, and strong evidence that night shift work exhibits key characteristics of carcinogens [3]. National Toxicology Program (NTP) concluded that there is sufficient evidence of carcinogenicity between night shift work exposure and breast cancer, based on the collective body of cancer epidemiology and mechanistic studies in humans [4]. There are many studies supporting the biological background of this association [5]. Detailed potential biological mechanistic connections between night shift work and breast cancer are provided in Supplementary texts A.

Furthermore, many previous studies on the association between light exposure at night and breast cancer support a positive association between night shift work and breast cancer because the biological background mechanisms for these two topics are similar [6–8]. A detailed explanation for the rationale is provided in Supplementary texts B.

For the association between night shift work and breast cancer, several studies even reported an effect modification by the hormone receptor status of breast cancer. Detailed explanation is provided in Supplementary texts C.

The hypothesis of this study is that night shift work exposure can increase the risk of female breast cancer. To validate this hypothesis, the authors conducted a two-stage dose-response meta-analysis on this topic. Because of several methodologic flaws observed in previous meta-analysis studies, the authors strictly determined and applied the inclusion criteria and applied strict statistical principles to each process of the meta-analysis. Most importantly, the exposure dose for each risk estimate was clearly defined based on the texts of individual original articles. Finally, the authors applied a reliable two-stage dose-response meta-analysis methodology [9, 10]. Based on these efforts to improve the quality of this dose-response meta-analysis, this study could add to the existing body of evidence.

## Methods

### Literature search

A literature search was conducted by a medical librarian in the medical library of Inha University, Incheon, South Korea (information specialist Minji Kim commented on the acknowledgment section). The medical librarian searched PubMed, EMBASE, and the Cochrane Library on December 30th, 2022.

### Inclusion criteria and selection of articles

The inclusion criteria were as follows: (i) the article should deal with the relationship between night shift work and breast cancer. (ii) The exposure of interest (night shift work) should be stated clearly in the text. Articles dealing with working experience in jobs that could be associated with night shift work as exposure of interest without a clear statement of night shift work were excluded because these jobs could not include night shift work in some cases. Articles dealing with light exposure at night, disturbance of the sleep-wake cycle, or the level of melatonin hormone as the exposure of interest were all excluded. Night shift work should be dealt with as occupational exposure, whether it was dealt with as a categorical or continuous variable. Variations in night shift work definition in each study were separately summarized in a table and considered in the evidence synthesis. (iii) Quantitative analysis should be included. The results should be provided as an RR or OR in cohort or case-control studies, respectively. Hazard ratio (HR) in survival analyses can be interpreted as RR based on the following previous studies [11–13]. (iv) Literature written only in English was included. (v) Articles with only human subjects (not animal subjects) were included. (vi) For article type, only the original article was included. However, if all other criteria were met, a letter to the editor was also included after a careful examination. The abstract was excluded because research abstracts are usually published as full articles in academic journals after an academic conference. Including an abstract could cause a duplication of the original research data. (vii) The articles dealing with male breast cancer were excluded. Only articles dealing with female breast cancer were included. (viii) Several articles were included additionally after the screening and review of the bibliographies of essential articles.

The first and corresponding author, JM, and the third author, YM, conducted the selection process separately. After this process, two selection results were compared with each other, and these two authors discussed them. Under this discussion, a final selection result was decided.

### Exposure, outcome, and confounding aspect of each study

The authors carefully examined each study from the perspective of selection, exposure, and outcome biases and confounding. The results of this examination were summarized in separate tables. Based on these results, the overall reliability of a study was assessed.

### Data extraction

Risk estimates from each study were extracted to construct the dose-response meta-analysis dataset. For the construction of a dose-response relationship, each risk estimate for each dose category of night shift work exposure was extracted. The dose of interest in this study was the years of night shift work.

### Examination of publication bias

The existence of publication bias was examined using Begg’s funnel plot and Egger’s regression test. If Begg’s funnel plot shows an asymmetric shape, the existence of a publication bias was suspected. Egger’s regression test uses the precision and the standardized effect size of the effect estimate from a study as the independent variable and dependent variable, respectively [14]. If Egger’s regression test result shows a statistically significant result, the existence of a publication bias could be suspected. The statistically significant p-value for publication bias was set at 0.05.

For Egger’s regression test, only one representative effect estimate (for example, RR for cohort studies or OR for case-control studies) is needed for each study. Therefore, the authors applied the same two-stage dose-response meta-analysis method used in this study (will be explained in subsection 2.6) to each study separately [9, 10]. Then, the authors calculated a representative RR or OR of breast cancer for one year increase in night shift work from each study. These RRs or ORs and calculated variance were used to conduct Egger’s regression test.

### Dose-response meta-analyses

For the investigation of the dose-response relationship between the years of night shift work (exposure dose) and the incidence of breast cancer (response), a two-stage dose-response meta-analysis was applied [9, 10].

First, the authors calculated a point dose for each dose range of exposure (years of night shift work). For a finite range with a lower and upper limit, we applied the median value for the range. For the highest dose range category, we added the half value of the interval for other dose ranges to the lower limit of the highest category. For example, if the dose ranges are comprised of 0, 0–10, 10–20, 20–30, and > 30, we assigned 0, 5, 15, 25, and 35 (30+(10/2)) as the point doses.

Second, we applied the two-stage dose-response meta-analysis methodology [10]. This process is composed of two stages. The first stage is to estimate the dose-response association between the adjusted log risk ratios and the levels of a specific exposure (point dose) in a particular study. The linear regression model is.

1 $$\text{y}=\text{X}\beta+\varepsilon$$

where the dependent variable y is an *n*×1 vector of log relative risks (not including the reference one), and X is a n×p matrix containing the non-referent values of the dose and/or some transforms of it (e.g., splines, polynomials). The variance-covariance matrix COV(ε) is equal to the following symmetric matrix.

2 $$\text{COV}(\varepsilon)=\text{S} =\left[\begin{array}{ccccc}{\sigma\:}_{1}^{2}&\:&\:&\:&\:\\\:⋮&\:\ddots\:&\:&\:&\:\\\:{\sigma\:}_{i1}&\:&\:{\sigma\:}_{i}^{2}&\:&\:\\\:⋮&\:&\:&\:\ddots\:&\:\\\:{\sigma\:}_{n1}&\:\cdots\:&\:{\sigma\:}_{ni}&\:\cdots\:&\:{\sigma\:}_{n}^{2}\end{array}\right]$$

where the covariance among (log) risk ratios implies the non-diagonal elements of S are unlikely to be equal to zero. For the covariance approximation, the authors applied a method devised by Greenland and Longnecker. The second stage is to combine study-specific estimates for the estimation of the trend. Each study included in the dose-response meta-analysis can be expressed as.

3 $$\:{\widehat{\beta\:}}_{j}\:\sim\:{N}_{p}(\beta\:,{\widehat{V}}_{j}+\psi\:)$$

where $\:{\widehat{V}}_{j}+\psi\:$ = $\:{\varSigma\:}_{j}$. The marginal model defined in Eq. 3 has independent within-study and between-study components. In the between-study components, $\:{\beta\:}_{j}$ is assumed to be sampled from $\:{N}_{p}(\beta\:,\psi\:)$, where ψ is the unknown between-study covariance matrix. Here, β can be interpreted as the population-average outcome parameters, namely the coefficients defining the pooled dose-response trend. The prediction of interest in a dose-response analysis is the relative risk for the disease comparing two exposure values. Given a range of exposure x and a chosen reference value $\:{x}_{ref}$, the predicted pooled dose-response association can be obtained as follows

4 $$\:\widehat{{RR}_{ref}}\:=\:exp\left\{\right(X-{X}_{ref}\left)\widehat{\beta\:}\right\}$$

where X and $\:{X}_{ref}$ are the design matrices evaluated, respectively, in x and $\:{x}_{ref}$. A (1-α/2) % confidence interval for the predicted pooled dose-response curve is given by

5 $$\:\text{E}\text{x}\text{p}\left\{\text{l}\text{o}\text{g}\right({\widehat{RR}}_{ref})\:\mp\:\:{z}_{\alpha\:/2}diag{\left(\right(X-{X}_{ref}\left)\widehat{V}\right(\widehat{\beta\:}\left){(X-{X}_{ref})}^{T}\right)}^{1/2}\}$$

where $\:\widehat{V}\left(\widehat{\beta\:}\right)$ is the estimated covariance matrix of $\:\widehat{\beta\:}.$

A dose-response meta-analysis was conducted two times, using all studies and only reliable studies, respectively.

### Statistical software

For all statistical analyses, R software version 4.2.2 was used. For a dose-response meta-analysis, the R package ‘dosresmeta’ was used [10].

## Results

### Literature search and screening

**Fig. 1:**
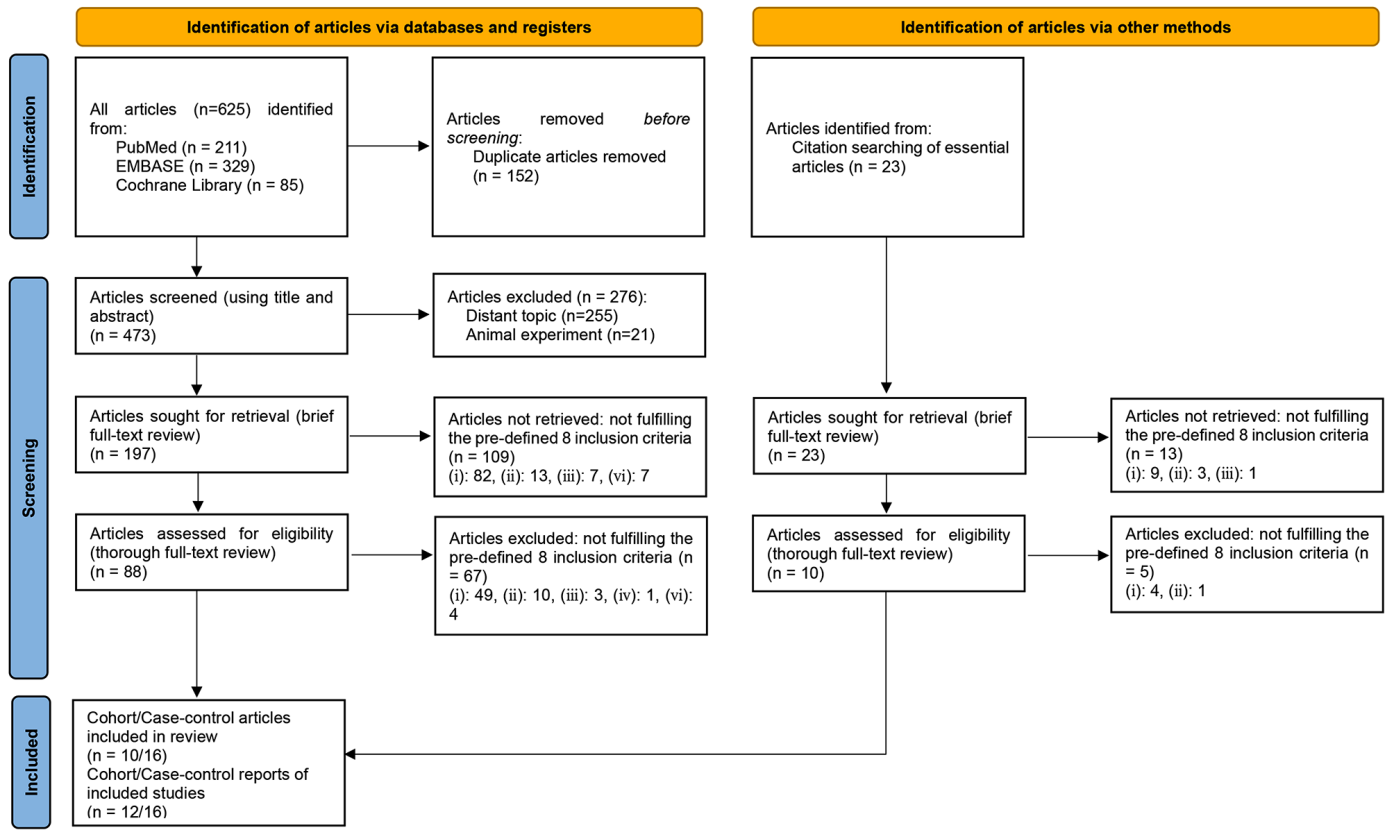
PRISMA flow diagram with grey literature [15]

Application form for systematic search is provided in Supplementary material B. Search terms and used syntax are provided in Supplementary material C. Search results in each of 3 databases are provided in Supplementary material D. The PRISMA flow diagram with grey literature is provided in Fig. 1. In the PubMed, EMBASE, and Cochrane Library, 211, 329, and 85 articles were searched, respectively. Among a total of these 625 articles, 152 articles were duplicated articles. Finally, 473 articles remained.

Researchers examined the title and abstract of 473 remaining articles. Among these remaining 473 articles, 276 articles were excluded (252 and 21 due to distant topics and animal subjects, respectively), and 197 candidate articles remained. For these 197 candidate articles, the original text was attached and examined (a brief full-text review). Through this step, 109 articles were excluded (82, 13, 7, and 7, due to inclusion criterion (i), (ii), (iii), and (vi), respectively), and 88 articles remained. For these remaining 88 articles, the authors conducted a thorough full-text review and strictly applied the pre-defined eight inclusion criteria for final selection. Through this step, 67 articles were excluded (49, 10, 3, 1, and 4 due to inclusion criteria (i), (ii), (ii), (iv), and (vi), respectively), and 21 articles remained.

Through a careful search of bibliographies of essential articles, the authors could find additional 23 candidate articles. For these 23 candidate articles, the original text was attached and examined (a brief full-text review). Through this step, 13 articles were excluded (9, 3, and 1 due to inclusion criteria (i), (ii), and (iii), respectively), and the other 10 articles remained. For these remaining 10 articles, researchers conducted a thorough full-text review and strictly applied the pre-defined eight inclusion criteria for final selection. Through this step, 5 articles were excluded (4 and 1 due to inclusion criteria (i) and (ii), respectively), and the other 5 articles remained.

Finally, 10 cohort study articles and 16 case-control study articles were included. For these 26 included articles, 12 cohort, and 16 case-control reports were included.

**Table 1:** Characteristics of cohort studies

Authors	Report distinction	Study year	Study country	Study name	Years of night shift work (range)	Years of night shift work (point dose)	Breast cancer cases (total cases: 18,086)	Person-years (total person-years: 7,987,557)	RR and 95% confidence interval (CI)
Akerstedt et al. (2015) [16]	1	1959, 1998–2003	Sweden	Screening Across the Lifespan Twin (SALT) study	0	0	354	84,163	1
					1–5	2.5	57	14,537	0.93 (0.66–1.31)
					6–10	7.5	16	5559	0.79 (0.45–1.38)
					11–20	15	18	5341	0.8 (0.45–1.42)
					21–45	32.5	18	2653	1.77 (1.03–3.04)
Jones et al. (2019) [17]	2	2003–2014	UK	Generation study	0	0	1845	249,864	1
					0–10	5	89	102,298	0.92 (0.74–1.14)
					10–20	15	65	192,878	1.09 (0.85–1.4)
					20–30	25	36	241,610	0.97 (0.7–1.35)
					> 30	35	24	347,826	1.12 (0.75–1.69)
Knutsson et al. (2013) [18]	3	1992–1995, 1996–1997, 2000–2003, 2009	Sweden	Work, Lipids, and Fibrinogen (WOLF) occupational cohort study	Day shifts	0	60	31,136	1
					Shift with night	Undetermined (9.39)	14	6807	2.02 (1.03–3.95)
Koppes et al. (2014) [19]	4	1996–2009	Netherlands	Dutch Labor Force Survey	0–3	1.5	684	768,825	1
					4–9	6.5	684	582,573	1 (0.9–1.12)
					10–19	15	708	413,986	1.07 (0.96–1.19)
					> 20	25	455	206,103	1.11 (0.98–1.26)
McNeil et al. (2020) [20]	5	2004, 2008, 2018	Canada	Alberta’s Tomorrow Project	0	0	258	101,376	1
					0.1–5.9	3	64	27,775	0.92 (0.7–1.21)
					> 6	9	45	16,879	1.02 (0.74–1.41)
Pronk et al. (2010) [21]	6	1996–2000, 2000–2002, 2002–2004, 2004–2007	China	Shanghai Women’s Health Study	0	0	423	370,476	1
					0–11	5.5	108	95,121	1.1 (0.9–1.3)
					11–22	16.5	89	99,504	0.9 (0.7–1.1)
					> 22	27.5	97	92,340	1 (0.8–1.3)
Sweeney et al. (2020) [22]	7	2003–2009	US	Sister study	0	0	2937	408,668	1
					0–5	2.5	88	9540	1.3 (1.05–1.61)
					5–10	7.5	30	5271	0.81 (0.57–1.16)
					> 10	12.5	42	5973	0.96 (0.71–1.31)
Travis et al. (2016): Million Women Study [23]	8	1996–2001, every 3–4 years, until 2009–2012	UK	Million Women Study	0	0	4136	1,170,603	1
					0–9	5	400	119,397	0.93 (0.83–1.03)
					10–19	15	140	33,152	1.14 (0.96–1.35)
					> 20	25	89	24,117	1 (0.81–1.23)
Travis et al. (2016): Epic-Oxford [23]	9	1993–1999	UK	Epic-Oxford study	0	0	153	59,795	1
					0–9	5	15	5638	1.18 (0.69–2.01)
					10–19	15	11	2185	1.92 (1.03–3.57)
					> 20	25	1	1475	0.22 (0.03–1.61)
Harma et al. (2022) [24]	10	1997, 2000, 2004, 2008, 2012	Finland	Finnish Public Sector study	0	0	10	8759	1
					5–9	7.5	4	10,587	0.69 (0.2–2.41)
					10–14	12.5	7	6066	1.48 (0.52–4.15)
					> 15	17.5	19	7483	1.65 (0.72–3.81)
Schernhammer et al. (2006): NHS2 [25]	11	1989	US	Nurses’ Health Study 2	0	0	441	426,119	1
					1–9	5	816	809,374	0.98 (0.87–1.1)
					10–19	15	80	72,829	0.91 (0.72–1.16)
					> 20	25	15	4881	1.79 (1.06–3.01)
Schernhammer et al. (2001): NHS1 [26]	12	1976, every 2 years, 1998	US	Nurses’ Health Study 1	0	0	925	298,815	1
					1–14	7.5	1324	383,882	1.08 (0.99–1.18)
					15–29	22.5	134	40,759	1.08 (0.9–1.3)
					> 30	37.5	58	12,559	1.36 (1.04–1.78)

**Table 2:** Characteristics of case-control studies

Authors	Report distinction	Study name	Study year	Study country	Years of night shift work (range)	Years of night shift work (point dose)	Cases (total 17,805 cases)	Controls (total 21,184 controls)	Total participants (total 38,989 participants)	OR and 95% CI
Bustamante et al. (2019) [27]	1	ISSEMyM Cancer Center study	Not mentioned	Mexico	No night shift work	0	68	97	165	1
					Night shift work	Undetermined (10.4)	33	4	37	8.58 (2.19–33.78)
Davis et al. (2001) [28]	2	Fred Hutchinson Cancer Research Center study	1992–1995	US	0	0	682	680	1362	1
					< 1.0	0.5	19	17	36	1.2 (0.6–2.3)
					1.0–3.0	2	20	15	35	1.4 (0.7–2.8)
					3.0-4.6	3.8	9	14	23	0.6 (0.3–1.5)
					> 4.6	5.4	33	15	48	2.3 (1.2–4.2)
Fritschi et al. (2013) [29]	3	Breast Cancer Employment and Environment Study	2009–2011	Australia	0	0	959	1476	2435	1
					< 10	5	140	160	300	1.35 (1.06–1.72)
					10–20	15	42	58	100	1.12 (0.74–1.68)
					> 20	25	24	40	64	0.96 (0.58–1.61)
Grundy et al. (2013) [30]	4	Vancouver and Kingston study	2005–2010	Canada	0	0	751	773	1524	1
					0–14	7.5	283	312	595	0.95 (0.79–1.16)
					15–29	22.5	72	81	153	0.93 (0.67–1.3)
					> 30	37.5	28	13	41	2.21 (1.14–4.31)
Hansen et al. (2001) [31]	5	Danish cancer study	Not mentioned	Denmark	daytime	0	5847	5723	11,570	1
					all night work combined	Undetermined (4.61)	434	301	735	1.5 (1.3–1.7)
Menegaux et al. (2013) [32]	6	CECILE study	2005–2007	France	0	0	1068	1170	2238	1
					< 4.5	2.25	66	69	135	1.12 (0.78–1.6)
					> 4.5	6.75	98	78	176	1.4 (1.01–1.92)
O’Leary et al. (2006) [33]	7	Long Island Breast Cancer Study Project	1996–1997	US	0	0	469	473	942	1
					< 8	4	11	16	27	0.74 (0.32–1.68)
					≥ 8	12	6	19	25	0.32 (0.12–0.83)
Papantoniou et al. (2016) [34]	8	MCC-Spain study	2008–2013	Spain	0	0	1438	1542	2980	1
					1–4	2.5	67	58	125	1.21 (0.83–1.76)
					5–14	10	103	85	188	1.13 (0.83–1.53)
					≥ 15	20	97	91	188	1.21 (0.89–1.65)
Pesch et al. (2010) [35]	9	GENICA study	2000–2004, 2004–2007	Germany	0	0	698	740	1438	1
					0–4	2.5	15	25	40	0.64 (0.34–1.24)
					5–9	7.5	11	12	23	0.93 (0.41–2.15)
					10–19	15	10	11	21	0.91 (0.38–2.18)
					≥ 20	25	12	5	17	2.49 (0.87–7.18)
Pham et al. (2019) [36]	10	South Korea NCC study	2012–2018	South Korea	0	0	1561	1541	3102	1
					< 10	5	139	145	284	1.07 (0.83–1.36)
					> 10	15	21	35	56	1.55 (0.89–2.69)
Wang et al. (2015) [37]	11	Guangzhou cancer study	2010–2012	China	night shift work: never	0	443	527	970	1
					night shift work: ever	Undetermined (10.0)	218	187	405	1.37 (1.07–1.74)
Yang et al. (2019) [38]	12	Jiujiang breast cancer study	2013–2016	China	night shift work: no	0	360	371	731	1
					night shift work: yes	Undetermined (10.0)	41	30	71	1.38 (1.04–2.71)
Hansen et al. (2012a): Danish military [39]	13	Danish military study	1990–2003	Denmark	0	0	88	361	449	1
					1-5.9	3.5	13	67	80	0.9 (0.4–1.7)
					6-14.9	10.5	18	48	66	1.7 (0.9–3.2)
					> 15	19.5	12	29	41	2.1 (1-4.5)
Lie et al. (2006) [40]	14	Norwegian nurse study	1960–1982	Norway	0	0	50	215	265	1
					0–14	7.5	362	1511	1873	0.95 (0.67–1.33)
					15–29	22.5	101	359	460	1.29 (0.82–2.02)
					≥ 30	37.5	24	58	82	2.21 (1.1–4.45)
Szkiela et al. (2021) [41]	15	Lodz region study	2015–2019	Poland	0	0	310	410	720	1
					1–9	5	19	17	36	1.48 (0.76–2.89)
					10–19	15	74	31	105	3.16 (2.02–4.92)
					20–29	25	44	20	64	2.91 (1.68–5.04)
					30–39	35	27	14	41	2.55 (1.32–4.95)
Hansen et al. (2012b): Danish nurse [42]	16	Danish nurse study	2001–2003	Denmark	Graveyard shifts: 0	0	37	252	289	1
					1–5	2.5	55	228	283	1.5 (0.99–2.5)
					5–10	7.5	70	195	265	2.3 (1.4–3.5)
					10–20	15	66	236	302	1.9 (1.1–2.8)
					≥ 20	25	39	124	163	2.1 (1.3–3.2)

### Characteristics of included studies

Table 1 provides the characteristics of 10 cohort studies that were included (12 reports). The total number of cases and person-years were 18,086 cases and 7,987,557 person-years, respectively. The study year for cohort construction starts in 1959, and the specific data extraction and analysis were conducted from 1992 to 2014. Three study reports were from the UK and the US, respectively, and another two study reports were from Sweden. One report was from the Netherlands, Canada, China, and Finland, respectively. One report applied dichotomous exposure classification, shift with night versus day shifts [18]. The longest range for years of night shift work was from 0 to 45 years, and the shortest range for years of night shift work was from 0 to > 10 years. The longest range for point dose (years of night shift work) was from 0 to 37.5 years, and the shortest range for point dose was from 0 to 9. The highest RR reported was 1.79 (95% CI 1.06–3.01) for the category of > 20 years compared to no night shift work in Schernhammer et al. (2006) [25]. The lowest RR reported was 0.69 (95% CI 0.20–2.41) for the category of 5–9 years compared to no night shift work in Harma et al. (2022) [24].

Table 2 provides the characteristics of the included 16 case-control studies (16 reports). The total number of cases, controls, and total participants was 17,805, 21,184, and 38,989, respectively. The study year ranged from 1960 to 1982 to 2015–2019. Two studies did not report the study year [27, 31]. Three studies were from Denmark. Two studies were from the US and China, respectively. One study was reported from Mexico, Australia, Canada, France, Spain, Germany, South Korea, Norway, and Poland, respectively. Four studies applied dichotomous exposure classification, night shift work versus no night shift work [27, 31, 37, 38]. The longest range for years of night shift work was from 0 to 30–39 years, and the shortest range for years of night shift work was from 0 to > 4.5 years. The longest range for point dose (years of night shift work) was from 0 to 37.5 years, and the shortest range for point dose was from 0 to 5.4 years. The highest OR reported was 8.58 (95% CI 2.19–33.78) for the category of ‘night shift work’ compared to ‘no night shift work’ in Bustamante et al. (2019) [27]. The lowest OR reported was 0.32 (95% CI 0.12–0.83) for the category of ≥ 8 years compared to no night shift work in O’Leary et al. (2006) [33].

Supplementary material E provides point dose estimates (years of night shift work) for studies with dichotomous exposure classification, night shift work versus no night shift work. The rationale for these calculations is co-provided (main texts and calculation).

### Exposure, outcome, and confounding aspects of each study

Supplementary material F summarized the selection, exposure, outcome, and confounding aspects of each study, which were extracted from the main texts of each study. Table 3 and 4 provide a summary table of special features for each study from the perspective of exposure, outcome, and confounding. In addition, the overall reliability of each study is provided in the rightmost column of these tables. Table 3 and 4 are for cohort and case-control studies, respectively. For selection bias, all studies did not have any special features related to this aspect. Therefore, we omitted the column for selection bias in Table 3 and 4.

For cohort studies, two studies were rated unreliable. Jones et al. (2019) was rated unreliable because of incomplete exposure assessment for night shift work that ended before the last 10-year period from the start of the study [17]. Knutsson et al. (2013) was rated unreliable because of only dichotomous categorization for night shift work: ‘shift with night versus day [18]. The total number of cases and person-years for reliable studies were 15953 cases and 6812138 person-years, respectively.

Five case-control studies were rated unreliable. Bustamante et al. (2019) was rated unreliable because of only dichotomous exposure categorization: night shift work versus no night shift work [27]. Fritschi et al. (2013) was rated unreliable because the exposure assessment for night shift work was rather simple. The main exposure of interest was sleep patterns and not night shift work [29]. Hansen et al. (2001) was rated unreliable because of only dichotomous categorization: all night work combined versus daytime work [31]. Wang et al. (2015) was rated unreliable because of only dichotomous categorization of night shift work exposure: ever versus never night shift worker [37]. Yang et al. (2019) was rated unreliable because of only dichotomous exposure categorization: ever versus never night shift worker [38]. The total number of cases, controls, and total participants for reliable studies was 9196, 12,210, and 21,406, respectively.

**Table 3:** Exposure, outcome, and confounding aspect of each study (cohort studies)

Authors	Report distinction	Exposure (definition of night shift work, assessment method)	Outcome (definition of breast cancer)	Confounding	Overall reliability
Akerstedt et al. (2015) [16]	1	Word definition ‘worked night’	Incident breast cancer	Typical covariates	Reliable
Jones et al. (2019) [17]	2	10 pm to 7 am Detailed questionnaires Incomplete exposure assessment for night shift work that ended before the last 10-year period*	Incident breast cancer	Typical covariates Time-varying covariates	Unreliable*
Knutsson et al. (2013) [18]	3	22:00–06:00 Only dichotomous categorization: shift with night versus day*	Incident breast cancer	Only two confounding variables in the final model: number of children and alcohol intake (However, after careful statistical examination)	Unreliable*
Koppes et al. (2014) [19]	4	Midnight to 6 am	Admission due to breast cancer	Typical covariates	Reliable
McNeil et al. (2020) [20]	5	Word definition ‘Rotated with nights’ ‘Straight night shifts’	Incident breast cancer	Typical covariates	Reliable
Pronk et al. (2010) [21]	6	‘Starting work after 10 PM at least 3 times a month for over 1 year’	Incident breast cancer	Typical covariates	Reliable
Sweeney et al. (2020) [22]	7	Night: ≥1 h between 12:00– 2:00 AM Rotating shift	Incident breast cancer (including ductal carcinoma in situ)	Typical covariates	Reliable
Travis et al. (2016): Million Women Study [23]	8	Midnight to 6:00 At least 3 nights per month	Incident breast cancer (invasive) and death due to breast cancer	Typical covariates	Reliable
Travis et al. (2016): Epic-Oxford [23]	9	Word definition ‘night shifts’ ‘at least one night per month or 12 nights per year’	Incident breast cancer (invasive) and death due to breast cancer	Typical covariates	Reliable
Harma et al. (2022) [24]	10	Recent definition of the IARC working group for night shift work	Incident breast cancer	Typical covariates	Reliable
Schernhammer et al. (2006): NHS2 [25]	11	Word definition ‘rotating night shifts for at least 3 nights per month’	Incident breast cancer	Typical covariates	Reliable
Schernhammer et al. (2001): NHS1 [26]	12	Word definition ‘rotating night shifts for at least 3 nights per month’	Incident breast cancer	Typical covariates	Reliable

*The main reason why the study was rated ‘unreliable’

**Table 4:** Exposure, outcome, and confounding aspect of each study (case-control studies)

Authors	Report distinction	Exposure (definition of night shift work, assessment method)	Outcome (definition of breast cancer)	Confounding	Overall reliability
Bustamante et al. (2019) [27]	1	9 pm to 7 am At least one year Only dichotomous categorization: night shift work vs. no night shift work*	Incident breast cancer	Typical covariates	Unreliable*
Davis et al. (2001) [28]	2	‘graveyard shift’ Specific start and stop times in defining each shift	Incident breast cancer	Typical covariates	Reliable
Fritschi et al. (2013) [29]	3	Midnight to 5 am: graveyard shift Rather simple: the main focus is not night shift work, but sleep pattern.*	Incident invasive breast cancer, excluding ductal carcinoma in situ	Typical covariates (severely simple)	Unreliable*
Grundy et al. (2013) [30]	4	23:00 to 7:00 Night shift jobs	Incident in situ or invasive breast cancer	Typical covariates	Reliable
Hansen et al. (2001) [31]	5	Not mentioned ‘night time work’ Only dichotomous categorization: all night work combined vs. day time work*	Incident breast cancer	Typical covariates (rather simple)	Unreliable*
Menegaux et al. (2013) [32]	6	11:00 pm to 5:00 am Night shift (overnight)	Incident breast cancer	Typical covariates	Reliable
O’Leary et al. (2006) [33]	7	Overnight shift 7:00 pm to the following morning	Incident in situ or invasive breast cancer	Typical covariates	Reliable
Papantoniou et al. (2016) [34]	8	Not mentioned ‘shift type (day, night, rotating)’	Incident breast cancer	Typical covariates	Reliable
Pesch et al. (2010) [35]	9	24:00 to 5:00 night shifts	Incident breast cancer	Only three confounding variables in the final model: family history of breast cancer, hormone therapy use, and number of mammograms (However, after careful statistical examination)	Reliable
Pham et al. (2019) [36]	10	09:00 pm to 08:00 am Night shifts regularly for at least 2 months	Incident breast cancer	Typical covariates	Reliable
Wang et al. (2015) [37]	11	Midnight to 06:00 am Night shift work Only dichotomous categorization: ever vs. never night shift worker*	Incident breast cancer	Typical covariates	Unreliable*
Yang et al. (2019) [38]	12	Not mentioned Only dichotomous categorization: ever vs. never night shift worker*	Incident invasive breast cancer	Typical covariates	Unreliable*
Hansen et al. (2012a): Danish military [39]	13	17:00 to 09:00 Night shift work	Incident breast cancer	Typical covariates	Reliable
Lie et al. (2006) [40]	14	Rotating night shifts Nurse study	Incident breast cancer	Only two confounding variables in the final model: employment time as a nurse and parity	Reliable
Szkiela et al. (2021) [41]	15	Word definition ‘night shift’	Incident breast cancer	Typical covariates	Reliable
Hansen et al. (2012b): Danish nurse [42]	16	Graveyard shifts: 19:00 to 09:00	Incident breast cancer	Typical covariates	Reliable

*The main reason why the study was rated ‘unreliable’

### Publication bias

**Fig. 2:**
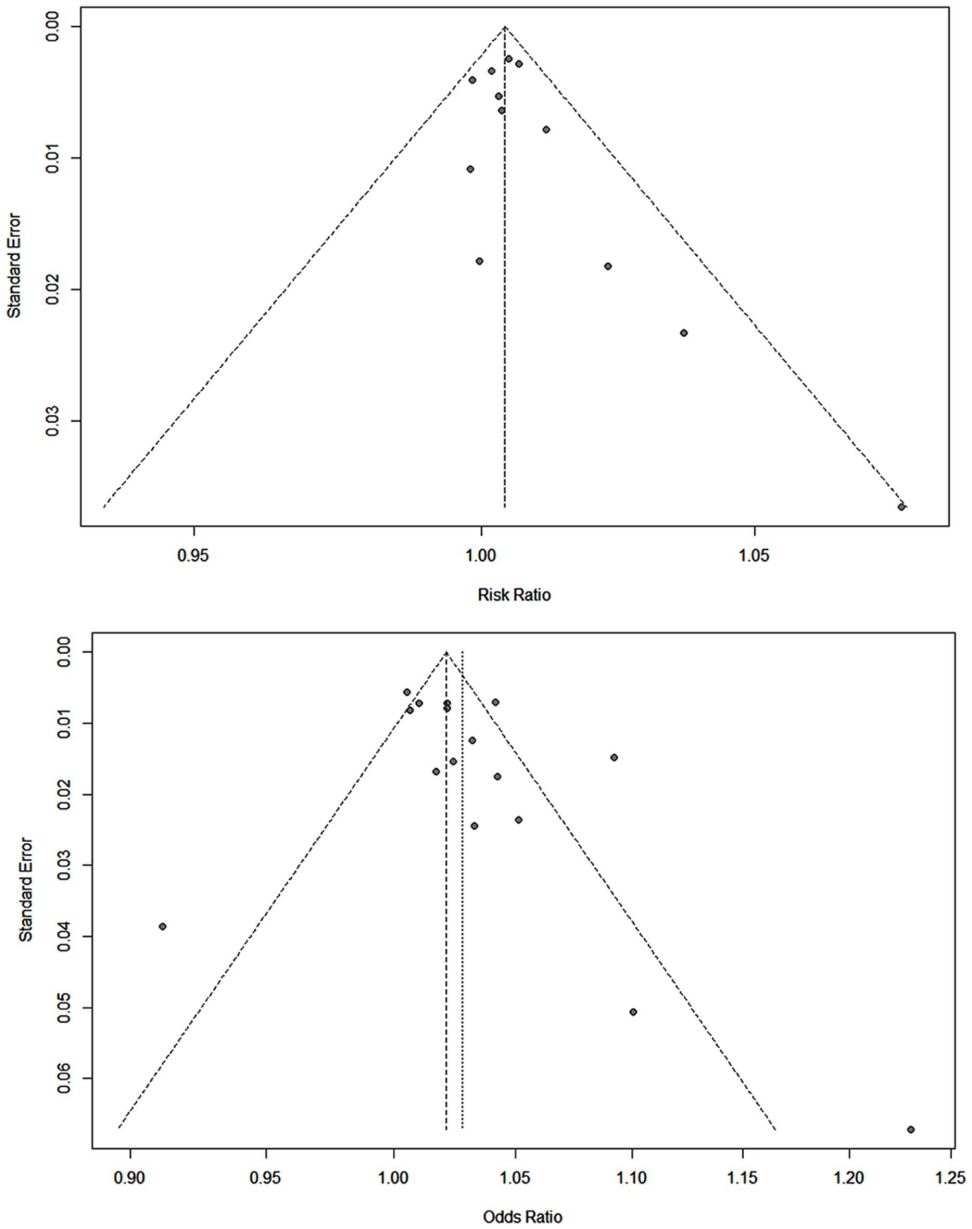
Begg’s funnel plot for cohort and case-control studies

Figure 2 provides each Begg’s funnel plot for cohort studies and case-control studies, respectively. The points in each funnel plot showed a relatively symmetric distribution (low possibility of publication bias). Supplementary material G provided the representative RRs and ORs calculated from each study using the same dose-response meta-analysis method used in this study. The Egger’s regression test result for cohort studies and case-control studies showed a p-value of 0.1437 and 0.1430, respectively. Based on these results, we concluded that the possibility of publication would be low.

### Dose-response meta-analysis

**Table 5:** Results of dose-response meta-analyses for only reliable studies

Study type	Years of night shift work			
	1 year	10 years	20 years	30 years
	Pooled RR (cohort studies) and pooled OR (case-control studies) for female breast cancer (95% CI)			
Cohort studies	1.0042 (1.0014–1.0070)	1.0425 (1.0138–1.0719)	1.0867 (1.0278–1.1490)	1.1328 (1.0419–1.2317)
Case-control studies	1.0213 (1.0108–1.0319)	1.2346 (1.1129–1.3695)	1.5242 (1.2386–1.8756)	1.8817 (1.3784–2.5687)

**Fig. 3:**
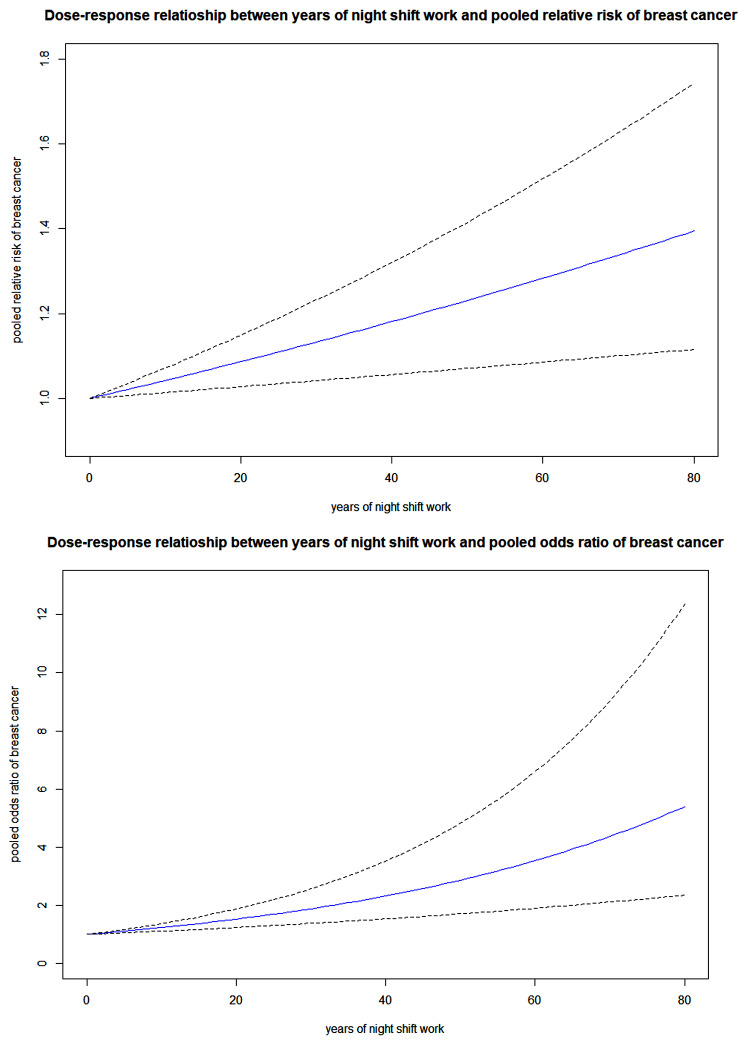
Dose-response meta-analysis plot for only reliable cohort (upper figure) and case-control studies (lower figure). The blue line is a continuous line of risk estimates along the increasing exposure (years of night shift work). Dashed lines are upper and lower bounds of 95% confidence intervals for risk estimates. Model: two-stage dose-response meta-analysis [9, 10]

Table 5 Provides the results of the dose-response meta-analyses for only reliable studies. First, the dose-response meta-analysis model for cohort studies and case-control studies was statistically significant with a p-value of 0.0035 and 0.0001, respectively. The pooled RR of female breast cancer (from cohort studies) for 1, 10, 20, and 30 years of night shift work exposure was 1.0042 (95% CI 1.0014–1.0070), 1.0425 (95% CI 1.0138–1.0719), 1.0867 (95% CI 1.0278–1.1490), and 1.1328 (95% CI 1.0419–1.2317), respectively. The pooled OR of female breast cancer (from case-control studies) for 1, 10, 20, and 30 years of night shift work exposure was 1.0213 (95% CI 1.0108–1.0319), 1.2346 (95% CI 1.1129–1.3695), 1.5242 (95% CI 1.2386–1.8756), and 1.8817 (95% CI 1.3784–2.5687), respectively. Figure 3 provides each dose-response meta-analysis plot for cohort studies and case-control studies, respectively, using only reliable studies. Supplementary material H provides the results of the dose-response meta-analyses for all studies and the dose-response meta-analysis plot for all cohort and case-control studies.

## Discussion

In this dose-response meta-analysis study, reliable 10 cohort and 11 case-control reports were included. Egger’s regression test result indicated that the possibility of publication bias is low. The risk of breast cancer increased by 0.42, 4.25, 8.67, and 13.28% after 1, 10, 20, and 30 years of night shift work exposure, respectively, according to cohort studies. The risk of breast cancer increased by 2.13, 23.46, 52.42, and 88.17% after 1, 10, 20, and 30 years of night shift work exposure, respectively, according to case-control studies.

### Review of previous meta-analyses

The following studies are recent systematic review and meta-analysis studies on the relationship between night shift work. Recent works are essential because they tried to synthesize all available evidence from individual articles published until the most recent days.

Van et al. concluded that night shift work is not associated with a risk of breast cancer [1]. In this study, only the case-control study group showed a statistically significant increased risk of breast cancer for ever night shift work versus never night shift work (pooled OR of 1.34, 95% CI 1.17–1.53). The pooled risk estimate for the nested case-control study group (pooled OR of 1.14, 95% CI 0.89–1.46) and that for the cohort study group (pooled RR of 0.98, 95% CI 0.93–1.03) were statistically insignificant for ever night shift work versus never night shift work. However, this study treated OR and RR as the same risk estimate and combined these two different types of risk estimates without any valid conversion. As seen in our results, the ORs from case-control studies tend to be higher than the RRs from cohort studies. This difference is also distinct in this study. Strictly speaking, OR can be converted into RR if the non-exposed prevalence for each OR is given [43]. In addition, this study did not consider a dose-response relationship between the years of night shift work and the risk of breast cancer. Only one risk estimate was extracted from each study, and this single estimate was used to represent the individual study. However, in occupational health and environmental health, a correct exposure assessment and consideration of dose-response relationship are essential keys for correct risk definition. Contrary to this study, the authors applied a reliable two-stage dose-response meta-analysis methodology to utilize multiple exposure doses and corresponding risk estimates reported in each individual article.

Manouchehri et al. concluded the pooled RR and 95% CI of breast cancer for < 10 years of night shift work was 1.13 (95% CI 1.03–1.24), and that for ≥ 10 years of night shift work was 1.08 (95% CI 0.99–1.17) [2]. In particular, studies with high quality and those adjusted for reproductive factors and family history of breast cancer showed an increased risk of breast cancer for night shift workers with statistical significance. This study classified the period of night shift work exposure into two categories: <10 years and ≥ 10 years of night shift work. However, the ≥ 10 years group showed a statistically insignificant increased risk, and < 10 years group showed a statistically significant increased risk. The statistically insignificant result for ≥ 10 years of night shift work could be due to a healthy worker survivor effect.

Dun et al. also reported that night shift work is not associated with the risk of breast cancer (pooled OR of 1.009, 95% CI 0.984–1.033) in a meta-analysis study [44].

### Examination of publication bias in a two-stage dose-response meta-analysis

In this study, the authors conducted Egger’s regression test using the representative RRs or ORs calculated by applying the same dose-response meta-analysis method to the effect estimates from each individual study separately. This method was devised by the authors, and further discussion is needed on this methodology. The authors will publish a methodology paper on this methodology with various examples, including the one from this study.

### Strengths and limitations of this study

Until today, the papers and meta-analysis papers indicated somewhat incompatible results on the association between night shift work exposure and female breast cancer incidence. This was because of (i) non-strict and non-transparent application of inclusion criteria for meta-analysis, (ii) misunderstanding of some essential differences between effect estimates obtained from different types of study design (for example, RRs and ORs), (iii) inaccurate exposure assessment in original studies or the application of inaccurate exposure dose in the meta-analysis, and (iv) the primitive evidence synthesis methods applied for the investigation of a dose-response relationship. On the contrary, this study (i) strictly applied eight transparent inclusion criteria, and (ii) thoroughly separated RRs from cohort studies and ORs from case-control studies and synthesized each type of effect estimates separately. In addition, (iii) exposure dose (years of night shift work) was clearly defined in evidence synthesis based on the years of night shift work reported in individual studies. Finally, (iv) the authors applied a reliable two-stage dose-response meta-analysis method reported in recent literature [9, 10].

Another strength of this study is a thorough review of previous studies regarding selection, exposure, outcome, and confounding aspects. Based on this thorough review process, the authors carefully excluded unreliable two cohort and five case-control studies from the initially included studies. This process enhanced the reliability of the results.

On the contrary, this study also has several limitations. First, this study separated cohort studies from case-control studies. However, under the rare disease assumption, the ORs acquired from case-control studies can be regarded as RRs acquired from cohort studies for rare diseases such as breast cancer [43]. The decision to separate these two types of studies was based on relatively lower risk estimates acquired from cohort studies than those from case-control studies. In future studies, the reason why the risk estimates from case-control studies are generally higher than those from cohort studies should be investigated further, and the risk estimates from case-control studies should be incorporated to those from cohort studies. Second, even though the authors analyzed the selection, exposure, outcome, and confounding aspects of each study, there would have been a remaining inter-study variation in the dose-response meta-analysis. In the future, if a cohort study with a far larger number of participants was conducted, a more conclusive result could be ascertained. Third, there is a possibility of survivorship bias. For example, women with 30 years of night shift work history should have survived at least 30 years after the start of night shift work to be included in this study as a 30-year night shift worker. Because the studies included in this meta-analysis defined the outcome as the incidence of or admission due to breast cancer, other causes of death except for breast cancer could have introduced this bias if the incidence rate of breast cancer had been different between dead night shift workers due to other causes and study participants. In future studies, this possibility should be considered. Fourth, environmental factors such as exposure to environmental estrogens or comorbidities such as type 2 diabetes mellitus and obesity may have contributed to the overall risk of breast cancer. Even though each included study adjusted for various potential confounders, it might have been difficult to consider confounders such as environmental estrogens or the use of personal care products (containing hormone disruptors) in these previous studies. These potential confounders should be considered in future studies.

## Conclusion

The risk of breast cancer increased by 0.42, 4.25, 8.67, and 13.28% after 1, 10, 20, and 30 years of night shift work exposure, respectively, according to a dose-response meta-analysis of 10 reliable cohort studies. The risk of breast cancer increased by 2.13, 23.46, 52.42, and 88.17% after 1, 10, 20, and 30 years of night shift work exposure, respectively, according to a dose-response meta-analysis of 11 reliable case-control studies. This study has several strengths from the perspective of a dose-response meta-analysis: Strictly applied eight inclusion criteria, separately synthesized RRs from cohort studies and ORs from case-control studies, clearly defined exposure dose, years of night shift work for each risk estimate, a reliable dose-response meta-analysis methodology, and careful considering of selection, exposure, and outcome biases and confounder adjustment for each study. This careful consideration of potential biases and confounding led to the exclusion of unreliable two cohort and five case-control studies.

### Electronic supplementary material

Below is the link to the electronic supplementary material.

Supplementary Material 1

## Data Availability

All data generated or analyzed during this study are included in this published article.

## Declarations

### Registration information for meta-analysis

This dose-response meta-analysis was not registered. However, the authors can provide any information regarding the process of evidence synthesis upon request.

### Protocol information

The protocol for this dose-response meta-analysis was not prepared. However, the authors can provide any information regarding the process of evidence synthesis upon request.
