# Supplementary material for: Night shift work and female breast cancer: a two-stage dose-response meta-analysis for the correct risk definition
Source: BMC Public Health. 2024 Jul 31;24:2065. doi: 10.1186/s12889-024-19518-2 (PMC11293116; doi:10.1186/s12889-024-19518-2)
Supplement: Supplementary file 1 — Supplementary Material 1 [file 12889_2024_19518_MOESM1_ESM.pdf]

SUPPLEMENTARY MATERIALS

Supplementary material A. National Toxicology Program (NTP) Office of Health Assessment and Translation (OHAT) Risk of Bias (RoB) rating tool

selection bias	1. Was administered dose or exposure level adequately randomized?
	2. Was allocation to study groups adequately concealed?
	3. Did selection of study participants result in appropriate comparison groups?
confounding bias	4. Did the study design or analysis account for important confounding and modifying variables?
performance bias	5. Were experimental conditions identical across study groups?
	6. Were the research personnel and human subjects blinded to the study group during the study?
attrition/exclusion bias	7. Were outcome data complete without attrition or exclusion from analysis?
detection bias	8. Can we be confident in the exposure characterization?
	9. Can we be confident in the outcome assessment?
selective reporting bias	10. Were all measured outcomes reported?
other bias	11. Were there no other potential threats to internal validity (e.g., statistical methods were appropriate and researchers adhered to the study protocol)?

Supplementary material B. Application form for systematic search

**Applicant**	Jinyoung Moon			**Application Date**	December 26 2022
**Topic**	The relationship between night-shift work and breast cancer: a dose-response meta-analysis				
**Keywords**	meta-analysis; dose-response meta-analysis; dose-response; dose-response relationship; breast cancer, breast tumor; breast malignancy<br>night-shift work, shift work, shift workers; work at night; night work; sleep duration; sleep habit;cohort study; case-control study; ecological study;				
**Target Databases**	PubMed / EMBASE / Cochrane Library				
**Languages**	English	**Search Period**	Until today	**Article Type**	No restriction
**P (Population, Patient)**	night-shift worker, people who work at night, shift worker				
**I (Intervention)**					
**C (Comparison, Control)**	day time worker, people who work at day time, regular worker				
**O (Outcome)**	mortality, incidence or prevalence of breast cancer, breast tumor, breast malignancy				
**Research Type**	No restriction			**Research Subject**	Not animal experiment
**References**	1. Van NTH, Hoang T, Myung S-K. Night shift work and breast cancer risk: a meta-analysis of observational epidemiological studies. Carcinogenesis. 2021. 2. Keyword : epidemiologic studiesshift workbreast cancer riskembase<br>2. Kamdar BB, Tergas AI, Mateen FJ, Bhayani NH, Oh J. Night-shift work and risk of breast cancer: a systematic review and meta-analysis. Breast Cancer Research and Treatment. 2013;138(1):291-301.				

Supplementary material C. Search terms and syntax

<table>
<tr><th rowspan="2">Keywords</th><th colspan="3">The selection of search terminology</th></tr>
<tr><th>MeSH</th><th>PubMed Entry Terms</th><th>Text Words</th></tr>
<tr><td rowspan="2">(P)
night-shift work</td><td>“Shift Work Schedule”[Mesh]</td><td>• Schedule, Shift Work
• Schedules, Shift Work
• Work Schedule, Shift
• Night Shift Work
• Shift Work, Night
• Rotating Shift Work
• Shift Work, Rotating</td><td>Evening shift</td></tr>
<tr><td>“Work Schedule Tolerance “[Mesh]</td><td>• Schedule Tolerance, Work
• Schedule Tolerances, Work
• Tolerance, Work Schedule
• Tolerances, Work Schedule
• Work Schedule Tolerances</td><td>Graveyard shift
Swing shift</td></tr>
<tr><td>(O)
Breast cancer</td><td>“Breast Neoplasms”[Mesh]</td><td>• Breast Neoplasm
• Neoplasm, Breast
• Breast Tumors
• Breast Tumor
• Tumor, Breast
• Tumors, Breast
• Neoplasms, Breast
• Breast Cancer
• Cancer, Breast
• Mammary Cancer
• Cancer, Mammary
• Cancers, Mammary
• Mammary Cancers
• Malignant Neoplasm of Breast
• Breast Malignant Neoplasm
• Breast Malignant Neoplasms
• Malignant Tumor of Breast
• Breast Malignant Tumor
• Breast Malignant Tumors
• Cancer of Breast
• Cancer of the Breast
• Mammary Carcinoma, Human
• Carcinoma, Human Mammary
• Carcinomas, Human Mammary
• Human Mammary Carcinomas
• Mammary Carcinomas, Human
• Human Mammary Carcinoma
• Mammary Neoplasms, Human
• Human Mammary Neoplasm
• Human Mammary Neoplasms
• Neoplasm, Human Mammary
• Neoplasms, Human Mammary
• Mammary Neoplasm, Human
• Breast Carcinoma
• Breast Carcinomas
• Carcinoma, Breast
• Carcinomas, Breast</td><td>Breast malignancy</td></tr>
</table>

Supplementary material D. Search results

Database 1: PubMed

Database	Search No.	Search String	Number of search results
PubMed	#1	“Shift Work Schedule”[Mesh]	995
	#2	“Shift Work Schedule”[TW] OR “Schedule, Shift Work”[TW] OR “ Schedules, Shift Work”[TW] OR “Work Schedule, Shift”[TW] OR “Night Shift Work”[TW] OR “Shift Work, Night”[TW] OR “Rotating Shift Work “[TW] OR “Shift Work, Rotating”[TW] OR “Evening shift”[TW]	2,072
	#3	“Work Schedule Tolerance”[Mesh]	7,453
	#4	“Work Schedule Tolerance”[TW] OR “Schedule Tolerance, Work”[TW] OR “Schedule Tolerances, Work”[TW] OR “Tolerance, Work Schedule”[TW] OR “Tolerances, Work Schedule”[TW] OR “Work Schedule Tolerances”[TW] OR “Graveyard shift”[TW] OR “Swing shift”[TW]	7,950
	#5 Combine	(((“Shift Work Schedule”[MeSH Terms]) OR (“Shift Work Schedule”[TW] OR “Schedule, Shift Work”[TW] OR “ Schedules, Shift Work”[TW] OR “Work Schedule, Shift”[TW] OR “Night Shift Work”[TW] OR “Shift Work, Night”[TW] OR “Rotating Shift Work “[TW] OR “Shift Work, Rotating”[TW] OR “evening shift”[TW])) OR (“Work Schedule Tolerance”)) OR (“Work Schedule Tolerance”[TW] OR “Schedule Tolerance, Work”[TW] OR “Schedule Tolerances, Work”[TW] OR “Tolerance, Work Schedule”[TW] OR “Tolerances, Work Schedule”[TW] OR “Work Schedule Tolerances”[TW] OR “graveyard shift”[TW] OR “swing shift”[TW])	9,235
	#6	“Breast Neoplasms”[Mesh]	334,775
	#7	((“Breast Neoplasm”[TW]) OR (“Neoplasm, Breast”[TW])) OR (“Breast Tumors”[TW])) OR (“Breast Tumor”[TW])) OR (“Tumor, Breast”[TW])) OR (“Tumors, Breast”[TW])) OR (“Neoplasms, Breast”[TW])) OR (“Breast Cancer”[TW])) OR (“'Cancer, Breast”[TW])) OR (“Mammary Cancer”[TW])) OR (“Cancer, Mammary”[TW])) OR (“Cancers, Mammary”[TW])) OR (“Mammary Cancers”[TW])) OR (“Malignant Neoplasm of Breast”[TW])) OR (“Breast Malignant Neoplasm”[TW])) OR (“Breast Malignant Neoplasms”[TW])) OR (“Malignant Tumor of Breast”[TW])) OR (“Breast Malignant Tumor”[TW])) OR (“Breast Malignant Tumors”[TW])) OR (“Cancer of Breast”[TW])) OR (“Cancer of the Breast”[TW])) OR (“Mammary Carcinoma, Human”[TW])) OR (“Carcinoma, Human Mammary”[TW])) OR (“Carcinomas, Human Mammary”[TW])) OR (“Human Mammary Carcinomas”[TW])) OR (“Mammary Carcinomas, Human”[TW])) OR (“Human Mammary Carcinoma”[TW])) OR (“Mammary Neoplasms, Human”[TW])) OR (“Human Mammary Neoplasm”[TW])) OR (“Human Mammary Neoplasms”[TW])) OR (“Neoplasm, Human Mammary”[TW])) OR (“Neoplasms, Human Mammary”[TW])) OR (“Mammary Neoplasm, Human”[TW])) OR (“Breast Carcinoma”[TW])) OR (“Breast Carcinomas”[TW])) OR (“Carcinoma, Breast”[TW])) OR (“Carcinomas, Breast”[TW])	352,512

Database	Search No.	Search String	Number of search results
	#8 Combine	(“Breast Neoplasms”[MeSH Terms]) OR (((“Breast Neoplasm”[TW]) OR (“Neoplasm, Breast”[TW])) OR (“Breast Tumors”[TW])) OR (“Breast Tumor”[TW])) OR (“Tumor, Breast”[TW])) OR (“Tumors, Breast”[TW])) OR (“Neoplasms, Breast”[TW])) OR (“Breast Cancer”[TW])) OR (“'Cancer, Breast”[TW])) OR (“Mammary Cancer”[TW])) OR (“Cancer, Mammary”[TW])) OR (“Cancers, Mammary”[TW])) OR (“Mammary Cancers”[TW])) OR (“Malignant Neoplasm of Breast”[TW])) OR (“Breast Malignant Neoplasm”[TW])) OR (“Breast Malignant Neoplasms”[TW])) OR (“Malignant Tumor of Breast”[TW])) OR (“Breast Malignant Tumor”[TW])) OR (“Breast Malignant Tumors”[TW])) OR (“Cancer of Breast”[TW])) OR (“Cancer of the Breast”[TW])) OR (“Mammary Carcinoma, Human”[TW])) OR (“Carcinoma, Human Mammary”[TW])) OR (“Carcinomas, Human Mammary”[TW])) OR (“Human Mammary Carcinomas”[TW])) OR (“Mammary Carcinomas, Human”[TW])) OR (“Human Mammary Carcinoma”[TW])) OR (“Mammary Neoplasms, Human”[TW])) OR (“Human Mammary Neoplasm”[TW])) OR (“Human Mammary Neoplasms”[TW])) OR (“Neoplasm, Human Mammary”[TW])) OR (“Neoplasms, Human Mammary”[TW])) OR (“Mammary Neoplasm, Human”[TW])) OR (“Breast Carcinoma”[TW])) OR (“Breast Carcinomas”[TW])) OR (“Carcinoma, Breast”[TW])) OR (“Carcinomas, Breast”[TW]))	438,486
	#9 Combine	#5 AND #8	257
	#10 Limit	#9 NOT (“animals”[MeSH])	211

Database 2: EMBASE

Database	Search No.	Search String	Number of search results
EMBASE	#1	“Shift Work Schedule”/exp	1,017
	#2	“Shift Work Schedule”:ti,ab,kw,hw OR “Schedule, Shift Work”:ti,ab,kw,hw OR “Schedules, Shift Work”:ti,ab,kw,hw OR “Work Schedule, Shift”:ti,ab,kw,hw OR “Night Shift Work”:ti,ab,kw,hw OR “Shift Work, Night”:ti,ab,kw,hw OR “Rotating Shift Work”:ti,ab,kw,hw OR “Shift Work, Rotating”:ti,ab,kw,hw OR “Evening shift”:ti,ab,kw,hw	2,065
	#3	“ Work Schedule Tolerance”/exp	9,945
	#4	“ Work Schedule Tolerance “:ti,ab,kw,hw OR “ Schedule Tolerance, Work “:ti,ab,kw,hw OR “ Schedule Tolerances, Work “:ti,ab,kw,hw OR “ Tolerance, Work Schedule”:ti,ab,kw,hw OR “ Tolerances, Work Schedule “:ti,ab,kw,hw OR “ Work Schedule Tolerances”:ti,ab,kw,hw OR “ Graveyard shift “:ti,ab,kw,hw OR “ Swing shift “:ti,ab,kw,hw	148
	#5 Combine	#1 OR #2 OR #3 OR #4	12,143
	#6	“Breast Neoplasms”/exp	625,217

Database	Search No.	Search String	Number of search results
	#7	“Breast Neoplasm”:ti,ab,kw,hw OR “Neoplasm, Breast”:ti,ab,kw,hw OR “Breast Tumors”:ti,ab,kw,hw OR “Breast Tumor”:ti,ab,kw,hw OR “Tumor, Breast”:ti,ab,kw,hw OR “Tumors, Breast”:ti,ab,kw,hw OR “Neoplasms, Breast”:ti,ab,kw,hw OR “Breast Cancer”:ti,ab,kw,hw OR “Cancer, Breast”:ti,ab,kw,hw OR “Mammary Cancer”:ti,ab,kw,hw OR “Cancer, Mammary”:ti,ab,kw,hw OR “Cancers, Mammary”:ti,ab,kw,hw OR “Mammary Cancers”:ti,ab,kw,hw OR “Malignant Neoplasm of Breast”:ti,ab,kw,hw OR “Breast Malignant Neoplasm”:ti,ab,kw,hw OR “Breast Malignant Neoplasms”:ti,ab,kw,hw OR “Malignant Tumor of Breast”:ti,ab,kw,hw OR “Breast Malignant Tumor”:ti,ab,kw,hw OR “Breast Malignant Tumors”:ti,ab,kw,hw OR “Cancer of Breast”:ti,ab,kw,hw OR “Cancer of the Breast”:ti,ab,kw,hw OR “Mammary Carcinoma, Human”:ti,ab,kw,hw OR “Carcinoma, Human Mammary”:ti,ab,kw,hw OR “Carcinomas, Human Mammary”:ti,ab,kw,hw OR “Human Mammary Carcinomas”:ti,ab,kw,hw OR “Mammary Carcinomas, Human”:ti,ab,kw,hw OR “Human Mammary Carcinoma”:ti,ab,kw,hw OR “Mammary Neoplasms, Human”:ti,ab,kw,hw OR “Human Mammary Neoplasm”:ti,ab,kw,hw OR “Human Mammary Neoplasms”:ti,ab,kw,hw OR “Neoplasm, Human Mammary”:ti,ab,kw,hw OR “Neoplasms, Human Mammary”:ti,ab,kw,hw OR “Mammary Neoplasm, Human”:ti,ab,kw,hw OR “Breast Carcinoma”:ti,ab,kw,hw OR “Breast Carcinomas”:ti,ab,kw,hw OR “Carcinoma, Breast”:ti,ab,kw,hw OR “Carcinomas, Breast”:ti,ab,kw,hw	684,807
	#8<br>Combine	#6 OR #7	697,013
	#9<br>Combine	#5 AND #8	363
	#10<br>Limit	Limit #9 to human / English language	329

Database 3: Cochrane Library

Database	Search No.	Search String	Number of search results
Cochrane Library	#1	[mh “Shift Work Schedule”]	37
	#2	(“Shift Work Schedule”):ti,ab,kw OR (“Schedule, Shift Work”):ti,ab,kw OR (“Schedules, Shift Work”):ti,ab,kw OR (“Work Schedule, Shift”):ti,ab,kw OR (“Night Shift Work”):ti,ab,kw OR (“Shift Work, Night”):ti,ab,kw OR (“Rotating Shift Work”):ti,ab,kw OR (“Shift Work, Rotating”):ti,ab,kw OR (“Evening shift”):ti,ab,kw	685
	#3	[mh “Work Schedule Tolerance”]	167
	#4	(“Work Schedule Tolerance”):ti,ab,kw OR (“Schedule Tolerance, Work”):ti,ab,kw OR (“Schedule Tolerances, Work”):ti,ab,kw OR (“Tolerance, Work Schedule”):ti,ab,kw OR (“Tolerances, Work Schedule”):ti,ab,kw OR (“Work Schedule Tolerances”):ti,ab,kw OR (“Graveyard shift”):ti,ab,kw OR (“Swing shift”):ti,ab,kw	254
	#5<br>Combine	#1 OR #2 OR #3 OR #4	827

Database	Search No.	Search String	Number of search results
	#6	[mh "Breast Neoplasms"]	14,921
	#7	("Breast Neoplasm"):ti,ab,kw OR ("Neoplasm, Breast"):ti,ab,kw OR ("Breast Tumors"):ti,ab,kw OR ("Breast Tumor"):ti,ab,kw OR ("Tumor, Breast"):ti,ab,kw OR ("Tumors, Breast"):ti,ab,kw OR ("Neoplasms, Breast"):ti,ab,kw OR ("Breast Cancer"):ti,ab,kw OR ("Cancer, Breast"):ti,ab,kw OR ("Mammary Cancer"):ti,ab,kw OR ("Cancer, Mammary"):ti,ab,kw OR ("Cancers, Mammary"):ti,ab,kw OR ("Mammary Cancers"):ti,ab,kw OR ("Malignant Neoplasm of Breast"):ti,ab,kw OR ("Breast Malignant Neoplasm"):ti,ab,kw OR ("Breast Malignant Neoplasms"):ti,ab,kw OR ("Malignant Tumor of Breast"):ti,ab,kw OR ("Breast Malignant Tumor"):ti,ab,kw OR ("Breast Malignant Tumors"):ti,ab,kw OR ("Cancer of Breast"):ti,ab,kw OR ("Cancer of the Breast"):ti,ab,kw OR ("Mammary Carcinoma, Human"):ti,ab,kw OR ("Carcinoma, Human Mammary"):ti,ab,kw OR ("Carcinomas, Human Mammary"):ti,ab,kw OR ("Human Mammary Carcinomas"):ti,ab,kw OR ("Mammary Carcinomas, Human"):ti,ab,kw OR ("Human Mammary Carcinoma"):ti,ab,kw OR ("Mammary Neoplasms, Human"):ti,ab,kw OR ("Human Mammary Neoplasm"):ti,ab,kw OR ("Human Mammary Neoplasms"):ti,ab,kw OR ("Neoplasm, Human Mammary"):ti,ab,kw OR ("Mammary Neoplasm, Human"):ti,ab,kw OR ("Breast Carcinoma"):ti,ab,kw OR ("Breast Carcinomas"):ti,ab,kw OR ("Carcinoma, Breast"):ti,ab,kw OR ("Carcinomas, Breast"):ti,ab,kw	43,822
	#8 Combine	#6 OR #7	43,822
	#9 Combine	#5 AND #8	134
	#10 Limit	#9 NOT ([mh "animals"])	85

Final search result

DBs	PubMed	EMBASE	Cochrane Library	Total including duplication	Duplicated	Total
Number of search results	211	329	85	625	152	473

Supplementary material E. Point dose estimate (years of night shift work) for studies with dichotomous exposure classification (night shift work versus no night shift work)

Study	Years of night shift work (point dose)	Main texts	Calculation
Knutsson et al. (2013) [1]	9.39	It was possible to calculate the exposure time for night work using responses from those who answered the WOLFU questionnaire [N=341, mean 9.39 years, standard deviation (SD) 9.53].	Mean = 9.39

Bustamante et al. (2019) [2]	10.4	Table 1<br>Characteristics: Years performing night work	101*0.148=14.948=15<br>101*0.178=17.978=18<br>Point dose<br>For 1-9 years of night shift work: (1+9)/2=5<br>For ≥10 years of night shift work: 10+5=15<br>5 years * 15 participants +15 years * 18 participants =345<br>345/(15+18)=10.4
Hansen et al. (2001) [3]	4.61	Thus, the risk of breast cancer among women with over 6 years of employment in trades with predominantly night work is 1.7 times that of daytime workers.	(0+6)/2=3 years<br>6+3=9 years<br>3 years * (434-117) = 951<br>9 years * 117 = 1053<br>951+1053 = 2004<br>2004/434=4.61
Wang et al. (2015) [4]	10	The authors arbitrarily applied 10 years of night shift work as the mean for all cases.	
Yang et al. (2019) [5]	10	The authors arbitrarily applied 10 years of night shift work as the mean for all cases.	

Supplementary material F. Selection, exposure, outcome, and confounding aspect of each study: extracted from the main text of each study

Cohort studies		
Source of heterogeneity	Akerstedt et al. (2015) [6]: Reliable	Special characteristic
Selection	Twins born in Sweden before 1959, who participated in the Screening Across the Lifespan Twin (SALT) study conducted by the Swedish Twin Registry (STR), and who at the time of the interview were 41–60 years old were included. Each individual participated in the SALT computer-assisted telephonic interview once between 1998 and March 2003. The response rate was 74%.<br>The exposed group was constituted of those who had worked at night for 1–45 years according to the response to the question: "For how many years have you had working hours that meant that you worked nights at least now and then".	Typical
Exposure	The exposed group was constituted of those who had worked at night for 1–45 years according to the response to the question: "For how many years have you had working hours that meant that you worked nights at least now and then". This group was compared with a group that had not worked nights. In addition, further categorisation of exposure was based on intervals in multiples of 5, that is, 1–5, 6–10, 11–20 or 21–45 years, in combination with observations that an effect may be expected for ≥30 years or ≥20 years. However, too few cases were obtained for categorisation at ≥30 years.	A few cases for the categorisation night shift work ≥30 years.
Outcome	Breast cancer was defined as having at least one new cancer diagnosis after the date of the interview, either according to the Cancer Register or to the Cause of Death Register.	Typical
Confounding	Educational level (0=compulsory (reference), 1=more than compulsory). Tobacco Use (0=no tobacco (reference), 1=tobacco use (includes current or previous regular smoking/taking snuff as well as occasional smoking or use of snuff)). Alcohol use (0=no alcohol consumption (reference), 1=alcohol consumption). Physical activity (0=moderate exercise (reference), 1=low exercise, 2=high exercise). (Question in SALT: "Of these 7 alternatives, which fits your annual exercise pattern?"). Body mass index (0=normal weight (>18.5–25 kg/m2; reference), 1=underweight (≤18.5), 3=overweight (>25–30), 4=obesity (>30)). Only one participant was underweight and	Typical

	was eliminated. Have children (0=no children (reference), 1=have children). Coffee use (1=no coffee (reference), 2=1–2 cups a day, 3=3–4 cups a day; 4=≥5 cups a day). Previous cancer (0=no cancer (reference), 1=have cancer) at the time of interview. Menopause (0=not passed (reference), 1=have passed) at the time of interview. Use of hormones, including oral contraceptives (0=no use (reference), 1=use) at the time of interview.	
Source of heterogeneity	Jones et al. (2019) [7]: Unreliable	Special characteristic
Selection	The Generations Study (GS) is a cohort study of >113,700 women aged 16 or older from the United Kingdom.<br>The first follow-up questionnaire was 2½ years after recruitment was completed by 99% of non-deceased participants, a second 6 years after recruitment by 97%, and a third 9½ years after recruitment by 96% of those recruited long enough ago to have entered this phase of follow-up.	Typical
Exposure	In relation to night shift work, women were asked in the recruitment questionnaire: "Over the last ten years, have you had any jobs that regularly involved work in the late evening or night (between 10 pm and 7 am)", and we collected information on type of job, year starting and ending, average number of nights per week working at night or late evening, and average number of hours worked between 10 pm and 7 am for each such episode of work. The same information on night work but covering the period from recruitment to the second follow-up questionnaire, was collected at this follow-up 6 years after recruitment. When analysing type of occupation in which night shift work occurred, if a woman reported different types of night work occupation concurrently during a time period, we counted only the type of work that she had done the most, so that we could allocate her to a single occupation at any one time. If the hours per night or nights per week of night work changed during a period of night work, we took an average of these night work intensity measures weighted by the number of years at each intensity. We did not ask about night shift work in the next follow-up questionnaire, 9½ years after recruitment.<br>To analyse breast cancer risk in relation to being a night shift worker in the last 10	Therefore, we were able to analyse comprehensive information on night shift exposures more than ten years ago that continued into the last 10-year period, but for exposures based on work history that ended before this 10-year period our analysis would be less complete.

	years, we updated night shift work status, and cumulative duration and time since cessation in single year increments, through to the 6-year follow-up. After this point, we assumed that women who had never been a night shift worker, or had ceased, did not commence new night work, and that women who were in current night work continued at the same intensity, frequency, and duration through to the end of analytic follow-up. Because our questionnaires only solicited information on night shift work history which, at least in part, had been undertaken in the last 10 years, we did not count information from night shift histories that ended completely >10 years ago (i.e., when women volunteered more information than requested) because we deemed this would be incomplete or missing for some women. Therefore, we were able to analyse comprehensive information on night shift exposures more than ten years ago that continued into the last 10 year period, but for exposures based on work history that ended before this 10 year period our analysis would be less complete.	
Outcome	Breast and other cancers occurring in the cohort were identified from recruitment and follow-up questionnaires, and spontaneous reports to the study centre. Spontaneous reports occurred when a woman contacted us and told us about her cancer diagnosis. For those lost to questionnaire follow-up, we ascertained cancers from linkage to National Health Service Central Registers (NHSCR), which provides information on vital status, cancer diagnosis and site. Confirmation of diagnosis was obtained from cancer registries in the United Kingdom, NHSCR linkage, pathology reports, and correspondence with patients' general practitioners.	Typical
Confounding	Information on risk factors for breast cancer was obtained from recruitment and follow-up questionnaires. Because we had collected ages or dates at which certain events or changes in lifestyle occurred, we were able to conduct analyses using time updated alcohol use, parity, oral contraceptive use, menopausal hormone therapy (MHT) use, and menopausal status, at the ages these events or changes occurred through to the second follow up questionnaire. We also updated post-menopausal body mass index (BMI) at the date of the second follow-up questionnaire.	Time-varying covariates were adjusted.
Source of heterogeneity	Knutsson et al. (2013) [1]: Unreliable	Special characteristic

Selection	The data were obtained from the WOLF (Work, Lipids, and Fibrinogen) occupational cohort study that included subjects who were employed in different public and private companies. The baseline study was first carried out in Stockholm, Sweden, from 1992–1995 (WOLFS, N=5698). In order to include more subjects, who worked in blue-collar jobs, a new data collection was carried out in two counties in the north of Sweden from 1996–1997 (WOLFN, N=4718). All subjects were employed at baseline (aged 19–70 years) and worked in 60 different companies.<br>The overall participation rate was around 80%. The subjects who participated in the 1996–1997 data collection were invited to a follow-up examination, which was performed during 2000–2003 (WOLFF, N=5433). Of those, 3630 were re-examined, and 1803 were recruited for the first time. In the present analysis, we included all women who entered the cohort in 1992–1995, 1996–1997, and 2000–2003, in total 4087 employees. Among those, it was possible to classify exposure to shift and day work among 4036 women, comprising the analytic sample for this study.<br>A new questionnaire was sent to all participants in 2009. In total, 2148 women answered the questionnaire in 2009 (WOLFU).	Typical
Exposure	Shift work<br>The two following questions were included in the WOLFS (1992–1995) and WOLFN (1996–1997) questionnaires: (i) “Do you work shifts?” Possible responses: (a) no; (b) 2 shifts; (c) 3 shifts with continuous operation; (d) according to a rota (irregular scheduling or working hours round the clock and over the whole week according to a particular work schedule; (e) other type of shift. (ii) “How many hours do you normally work per week including overtime, and how are these hours distributed on average?” Possible responses: (a) day work (06:00–18:00 hours), number of hours=xx; (b) evening work (18:00–22:00 hours), number of hours=xx; (c) night work (22:00–06:00 hours), number of hours=xx. In the WOLFF questionnaire (2000–2003), the questions were similar but the response options to question (i) were changed to: (a) no; (b) 2 shifts; (c) 3 shifts with continuous operation; (d) according to a rota without night shifts (irregular scheduling or working hours round the clock and over the whole week according to a particular work schedule); (e) according to a rota with night shifts; (f)	Only dichotomous categorization: shift with night versus day

	permanent night work; and (g) other type of shift.<br>In addition, the WOLFF questionnaire asked if the respondent had changed shift system during the last five years – either from shift to day or vice versa.<br>In the WOLFU questionnaire in 2009, the following questions were asked: (i) "Which option is best suited in terms of your current working hours? If you are not employed right now we want you to think of the time just before you stopped working?" (a) day work (about 06:00–18:00 hours); (b) evening work (about 18:00– 22:00 hours); (c) night work (about 18:00–06:00 hours); (d) shift work, not night; (e) shift work, including night; (f) according to a rota (ie, working hours according to a particular work schedule), not night; (g) according to a rota (ie, working hours according to a particular work schedule), including night; (h) other working hours. (ii) "Over how many years of your career have you worked shifts? If you have never worked shifts, enter 0 years." (iii) "How many years have included night work? If you have never worked shifts with night work, indicate the last year you worked shifts, and enter 0 years."<br>In order to categorize the participants in three groups (ie, day work and shift work with and without night shifts), we used data from baseline, follow up in 2000–2003 (WOLFF), and follow-up in 2009 (WOLFU). If data indicated day work on all occasions when the subject participated, she was regarded as a day worker. If data indicated shift work without night work on ≥1 occasion, and day work for the rest, the participant was defined as a worker with shift work without night work. If the data indicated shift work with night work on ≥1 occasion, and day work or shift work without night for the rest, the participant was regarded as a worker with night shift work. Information on shift/day work was obtained from 1459 participants from baseline only. In total, 2148 subjects participated in baseline and WOLFU, and 429 subjects participated on all three occasions (WOLFN or WOLFS, WOLFF and WOLFU).	
Outcome	Data on cancer incidence were obtained from the Swedish cancer registry from its establishment in 1958 to 2008. Reporting is mandatory and covers the total population. It includes individual data on personal identification number, sex, place of residence, site of tumor, histological type, basis and date of diagnosis, date and cause	Typical

	of death, date of migration, and whether a patient was registered as a resident in Sweden at the end of a specific year. However, cases without a cancer notification, but reported to the Cause of Death Register, are not included.<br>The date and causes of death were obtained from the Swedish death registry between 1992–2008. Women who developed breast cancer between 1958 and baseline were excluded from the analyses (N=10). All deceased women with breast cancer as an underlying cause in the death certificate (N=8), were included in the Swedish cancer registry in the present study.	
Confounding	The following were potential confounding variables from baseline: (i) body mass index (BMI) (continuous); (ii) waist-hip ratio (continuous); (iii) educational level dichotomized in two ways: educ1 (coded 1 for university or college, coded 0 for lower than university/college education), educ2 (coded 1 for high school or higher level, coded 0 for lower level than high school); (iv) current smoking (current versus not current smoker); (v) ever smoker (ever versus never smoker); (vi) menopausal status, and (v) treatment with hormones other than oral contraceptives.<br>All variables were not kept in the main model because they only marginally influenced the HR. Therefore, the final model included only exposure to day/shift work, number of children and alcohol intake.	Only two confounding variables were included in the final model.
Source of heterogeneity	Koppes et al. (2014) [8]: Reliable	Special characteristic
Selection	The study sample consisted of participants of the 14 Dutch Labor Force Surveys that were performed in the period 1996 until 2009.<br>Each year, Statistics Netherlands randomly sampled persons aged 15 years and older from the national household registers, except for persons living in institutions or collective households. The annual samples consisted of approximately 118,000 individuals per year, resulting in a total sample of 828,121 men and 829,039 women.<br>Women who were hospitalized with breast cancer in 1996 or later, but before or in the same month as their enrolment in the study were excluded from all analyses, to reduce the risk of reversed causality and recall bias, as breast cancer diagnosis may affect night work engagement and recall.	Typical

	Only women aged 15–64 years at the time of participating in the Labor Force Survey, and who had nonmissing data on night work were selected (Fig. 1).	
Exposure	Current exposure to night work was asked with the question: 'Do you work at nights, meaning between midnight and 6 am?'. The answering options were 'No', 'Yes, sometimes', and 'Yes, regularly'.	Typical
Outcome	The outcome variable of the present study was hospital admission due to breast cancer. The National Medical Registration (NMR) of the years 1996 through 2009 were used to identify women admitted to hospital due to breast cancer. In this NMR, hospital admissions for (part of) 1 day or more are registered, together with data on diagnosis and treatment. Almost all general and university hospitals, as well as several specialized hospitals in The Netherlands provide data to the NMR, which resulted in more than 95 % coverage in most of the years that were used for this study.	The outcome of interest was defined as hospital admission due to breast cancer.<br>The national database (NMR) cannot cover the whole population of the Netherlands (95% coverage).
Confounding	Analyses were performed both without and with adjustment for age, origin, children in the household, education, occupational group, contractual working hours, and job tenure. Women with missing data on one of these variables were excluded from all analyses to improve comparability of findings.	Typical
Source of heterogeneity	McNeil et al. (2020) [9]: Reliable	Special characteristic
Selection	Participants were recruited through 8 waves of telephone-based random digit dialing using regional health authority boundaries within the province of Alberta as the sampling frame, and a 2-stage method to identify individuals.	Typical
Exposure	Surveys 2004 and 2008 from Phase I of ATP collected information on rotating and night shift work employment and sleep duration. Participants were asked: "For how many years did you work a schedule that included day or evening work that rotated with nights in the same month?" and "For how many years did you work straight night shifts?" Participants who completed Survey 2004 were also asked: "On average, how many hours did you sleep each night during the past 4 weeks?", whereas participants who completed Survey 2008 were asked: "On average over the past 7 days, at what time did you normally go to sleep?" and "On average over the past 7 days, at what time did you normally wake up?" This information was used to estimate sleep duration.	Typical

Outcome	Data on primary incident cancers were obtained through linkage with the Alberta Cancer Registry (ACR) in August 2018. Incident cases of breast, prostate, colorectal and lung cancers were included in these analyses based on scientific plausibility and the number of cancer cases exceeding 100 cases/site.	Typical
Confounding	Covariates of interest included: age (years), sex (male/female), body mass index (<25 kg/m2/≥25 kg/m2), highest level of education (high school or less/some post-high school education or a post-high school certificate or degree), total household income ($0 to $49,999/$50,000 to $99,999/≥$100,000), employment status (yes/no), ethnicity (Caucasian/Other), marital status (married or living with someone/divorced, separated, or widowed/single, never married), smoking status (daily/occasional/former/never), presence of at least one medical condition/co-morbidity (e.g. diabetes, depression) (yes/no), and having a family history of cancer (yes/no). A proxy for menopausal status (<55 years of age/≥55 years of age) was included in the breast cancer incidence analysis.	Typical
Source of heterogeneity	Pronk et al. (2010) [10]: Reliable	Special characteristic
Selection	The Shanghai Women's Health Study is a population based prospective cohort study being conducted in Shanghai, China. All women aged 40–70 years in 7 representative urban communities of Shanghai identified through resident offices were eligible for inclusion. Between 1996 and 2000, 81,170 women were approached in person by trained interviewers, and 74,942 women were recruited, resulting in a participation rate of 92%. For the present analyses, women who had a history of cancer at study entry (n = 1,576), had never held a job outside the home (n = 274), or did not have complete data on work history (n = 43) were excluded.	Typical
Exposure	We assessed night-shift work by applying a job exposure matrix developed by an experienced Chinese industrial hygienist (S. X.) who was familiar with local industrial conditions. The job exposure matrix classified jobs in the occupational histories into 3 categories with increasing scores for night-shift work: 0 = no night-shift work; 1 = incidental night-shift work (e.g., reporters and army personnel); 2 = jobs likely to involve the night shift, but entailing working only part of the night or being on call	Typical

	(e.g., physicians and bakers); and 3 = jobs that probably involved all-night shifts (e.g., nurses, firemen, and certain production workers).<br>We computed lifetime number of years spent in jobs with possible night-shift work by summing the total number of years spent in jobs with a score higher than 0.<br>In addition, during the second follow-up (2002–2004), every participant was asked whether she had ever held a job involving night-shift work. Night-shift work was defined as starting work after 10 PM at least 3 times a month for over 1 year. Data on the average number of night shifts worked per week, the duration of shift work (years), and the years of starting and ending shift work were also collected. Cumulative night-shift work was calculated by multiplying the average number of night shifts per week by the duration of shift work.	
Outcome	The cohort was followed for the occurrence of cancer and other chronic diseases through a combination of biennial in-person interviews and annual record linkage to the Shanghai Cancer Registry and the Shanghai vital statistics database.<br>All possible incident cancer cases were verified through home visits. Medical charts from the diagnostic hospital were reviewed to verify the diagnosis.	Typical
Confounding	Presented hazard ratios were adjusted for age, education, family history of breast cancer, number of pregnancies, age at first birth, and occupational physical activity. Other potential confounders, including age at menarche, menopausal status, body mass index, calorie intake, and nonoccupational physical activity, were examined but were found not to alter the risk estimates meaningfully and therefore were not included in the final model.	Typical
Source of heterogeneity	Sweeney et al. (2020) [11]: Reliable	Special characteristic
Selection	The Sister Study is a prospective cohort of 50,884 women (2003–2009), ages 35–74 who had a sister with breast cancer but were breast cancer free themselves.	Typical
Exposure	Baseline occupational questionnaires asked about current job and past jobs held for ≥2 years. For each job, women were asked if they worked regular hours (starting/stopping at the same time every day) and if they worked at night (≥1 hour between 12:00– 2:00 AM). If they reported not working regular hours, they were asked if they worked	Typical

	rotating shifts (number of shifts and usual start/stop times for each shift) or irregular hours. Rotating shift work (N=3,183) included women who reported ≥1 job with rotating shifts; rotating night shift work (N=2,275) was a subset of those women who also reported night work for the same job. Working irregular hours (N=15,895) could include women who reported night work for ≥1 job. Any work at night (N=13,992) included all women, regardless of schedule type, who met our definition of night work for ≥1 job.	
Outcome	Self-reported incident breast cancer, confirmed with medical records, was classified as invasive breast cancer or ductal carcinoma in situ. Women were followed through September 15, 2017 (data release 7.2). The final sample included 48,451 women who completed the occupational questionnaire and were not missing covariate data.	Typical
Confounding	Confounders were selected a priori and included race/ethnicity, education, marital status, and parity. We explored whether the association differed by time-varying menopausal status, timing of starting work relative to first childbirth, or time since stopping shift work.	Typical
Source of heterogeneity	Travis et al (2016): Million Women Study [12]: Reliable	Special characteristic
Selection	During 1996-2001, a total of 1.3 million women aged 50-64 years who had been invited for routine screening for breast cancer at 66 screening centres in England and Scotland completed the recruitment questionnaire. The questionnaire asked about various characteristics, including anthropometric and reproductive factors, and other personal characteristics. Since recruitment, four questionnaires have been sent to the cohort participants approximately every 3-4 years to update and expand information on various factors.	Typical
Exposure	The fourth survey questionnaire was sent out in batches to the study population in 2009-12, and included questions for the first time about women's history of night shift work, as described previously.<br>Participants were therefore asked "Have you ever regularly worked at night or on night shifts (at any time between midnight and 06:00 hours, for at least 3 nights per month)?". Those who answered "yes" were asked about the duration ("Over how many years in	Typical

	total?") and timing of night shift work ("When did you last work at night?").	
Outcome	Participants from the Million Women Study are flagged on the NHS Central Registers so that cancer registrations and deaths can be routinely notified to the investigators. The Central Registers provide information on the date of each event and code the cancer site and cause of death according to the 10th revision of the International Classification of Diseases (ICD). The endpoints included in these analyses are first diagnosis of invasive breast cancer (ICD-10 C50) and death attributed to breast cancer (ICD-10 C50).	Typical
Confounding	Analyses were also additionally adjusted for the following characteristics (from the recruitment survey unless otherwise stated): quintiles of socioeconomic status (based on Townsend deprivation index for area of residence at recruitment) (7); age at menarche (<12, 12-13, 14 or more years), parity and age at first birth (nulliparous, 1-2, or 3 or more births cross-classified by age at first birth <25, ≥25 years), body mass index (<25, 25-30, ≥30 kg/m2), alcohol intake (0, 1-2, 3-6, 7-14, ≥15 drinks/week), smoking (never, past, current <15 cigarettes/day, current ≥15 cigarettes/day), strenuous physical activity (never/rarely, less than weekly, at least weekly), family history of breast cancer (yes, no), married/living with a partner (yes, no), use of oral contraceptives (never, ever), and HRT use (never, past, current). For each adjustment variable, missing values were assigned to a separate category.	Typical
Source of heterogeneity	Travis et al (2016): Epic-Oxford [12]: Reliable	Special characteristic
Selection	EPIC Oxford is a prospective cohort study of 63 429 women and men aged ≥20 years who were recruited from around the United Kingdom between 1993 and 1999.<br>Recruitment was through general practice surgeries and through postal recruitment, and participants completed a questionnaire, which asked about diet, anthropometry, lifestyle and reproductive factors, prior disease and other personal characteristics. Subsequently, follow-up questionnaires have been sent to the cohort participants approximately every 5 years to update and expand information on various factors.	Typical
Exposure	The fourth questionnaire was sent out in 2010 and included questions for the first time about individual's history of night shift work.	Typical

	Participants were therefore asked “Have you regularly worked at night, on night shifts or on call at night? (Please only consider any job lasting for at least one year, and occurring on a regular basis for at least one night per month or 12 nights per year)”. Those who answered “yes” were asked about the overall duration (“Over how many years in total?”) and timing of the night shift work (“When did you last work nights?”), before being asked to provide more detailed information about each job which had involved night shift work (“How many different jobs have you had involving night work?”) and for each were asked to provide information on nights per month, hours per night and shift pattern [fixed/permanent, rotating, flexible/irregular].	
Outcome	Participants from the EPIC-Oxford cohort were followed-up via record linkage to the NHS Central Registers, which provide information on cancer registrations and deaths, until the censoring date of December 31, 2013. The endpoints included in these analyses are first diagnosis of invasive breast cancer (ICD-10 C50) and death attributed to breast cancer (ICD-10 C50).	Typical
Confounding	Analyses were additionally adjusted for the following characteristics (from the recruitment survey unless otherwise stated): quintiles of socioeconomic status (based on Townsend deprivation index for area of residence) (7); age at menarche (<12, 12-13, ≥14 years), parity and age at first birth (nulliparous, or 1-2, ≥3 births cross-classified by age at first child <25, ≥25 years), body mass index (<20, 20<22.5, 22.5<25, 25<27.5, ≥27.5 kg/m2), alcohol intake (<1, 1-7, 8-15, ≥16 g/day), smoking (from the fourth survey; never, past, current <15 cigarettes/day, current ≥15 cigarettes/day), strenuous physical activity (never, <2 hours per week, ≥2 hours per week), married or living as married (yes, no), use of oral contraceptives (never, ever) and HRT use (never, ever). For each adjustment variable, missing values were assigned to a separate category.	Typical
Source of heterogeneity	Harma et al. (2022) [13]: Reliable	Special characteristic
Selection	The data for this study include those of the female members of the Finnish Public Sector (FPS) study. FPS is an ongoing dynamic survey cohort initiated in 1997 including several waves of questionnaire- based surveys between the years 1997 up to the present. The most common occupations in the FPS cohort are those related to healthcare, social services and education. In healthcare and social services, dominated	In order to improve exposure assessment for the long follow- up, the analysis of the whole sample was limited to participants with stable shift work status in two consecutive FPS surveys.

	by women and shift work, the most common job titles are nurse, practical nurse, department secretary, hospital cleaner and laboratory nurse.<br>The survey cohort (figure 1) comprises all female participants who had responded to any of the FPS questionnaires in 2000, 2004, 2008 and 2012 (response rates 66%–68%). In order to improve exposure assessment for the long follow- up, the analysis of the whole sample was limited to participants with stable shift work status in two consecutive FPS surveys.<br>Those shifting between any of the three categories of shift work status (day work, shift work without nights and shift work with nights) during the two surveys were excluded.	
Exposure	The participants were first classified as (1) day workers, (2) shift workers without night shifts, (3) shift workers with night shifts, (4) permanent night workers or (5) workers in 'other work schedules', based on a direct question or the presence of different work shifts. Those with 'other work schedules' or missing shift work status (n=884) were excluded. Shift work with night shifts and permanent night work were combined due to the low number (2%) of permanent night workers and based on the recent definition of the IARC working group for 'night shift work'.<br>The surveys of the hospital subcohort included an additional question of earlier shift work exposure ('how many years in total have you worked shift work?').	Typical
Outcome	Cases of breast cancer were retrieved from the Finnish Cancer Registry using the unique personal identification applied to all residents of Finland (www.cancerregistry.fi).<br>In the Finnish Cancer Registry, the cases have been registered according to the International Classification of Diseases for Oncology, Third Revision (ICD- O- 3) from 2007 onwards as well as the date of diagnosis. Cancers from 1953 to 2006 were coded by ICD- 7 and have been converted to ICDO- 3.	Typical
Confounding	For all shift work exposure variables, we computed the crude and adjusted HRs and 95% CIs, controlling for age, SES, children aged 0–6 years, children aged 7–18 years, smoking, alcohol consumption and BMI.	Typical
Source of heterogeneity	Schernhammer et al (2006): NHS2 [14]: Reliable	Special characteristic

Selection	The Nurses' Health Study II is a prospective cohort study that began in 1989, when 116,671 registered female U.S. nurses of ages 25 to 42 years were enrolled. Since 1989, they have completed biennial mailed questionnaires that include items about their health status and known or suspected risk factors for cancer. Response rates to questionnaires are at 90%.	Typical
Exposure	The 1989 questionnaire included detailed questions on total months during which study participants had worked on rotating night shifts for at least 3 nights per month in addition to having worked days or evenings in that month. This information was updated in 1991, 1993, and 1997. The prespecified categories for total numbers of months working on rotating night shifts were none, 1–4, 5–9, 10–14, 15–19, and 20 or more. Because the 1995 and 1999 questionnaires did not assess night work information, women were queried retrospectively in 2001 for the time periods 1993–1995 and 1997–1999. Questions were asked regarding both rotating night shifts, as previously described, and permanent night shifts for 6 or more months.	Typical
Outcome	We identified breast cancer cases as cases occurring between 1 June 1989 and 1 June 2001. Nurses who reported breast cancer were asked for permission to review their medical records, and breast cancer was confirmed through review of these records. In addition, approximately two thirds of the deaths among cohort members were reported to us by next of kin or the postal system in response to follow-up questionnaires. We also searched the National Death Index to identify deaths among the nonrespondents to each 2-year questionnaire.7 A total of 1,352 cases of breast cancer were reported in the base population during 12 years of follow up, and pathology records were obtained for 98%.	Typical
Confounding	Relative risk adjusted for age, age at menarche(<12, 12, 13, ≥14 yr), menopausal status (premenopausal, postmenopausal, unknown), age at menopause (<48, 48, 49, ≥50 yr), age at first birth and parity combined (nulliparous; age at first birth <25, 1–2 children; age at first birth 25–29, 1–2 children; age at first birth ≥30, 1–2 children; age at first birth <25, ≥3 children; age at first birth ≥25, ≥3 children), body mass index (weight in kilograms divided by the square of height in meters; <18.5, 18.5–19.9, 20.0–22.4, 22.5–24.9, 25.0–29.0, and ≥30 kg/m2), current alcohol consumption (nondrinkers, <5 g/d,	Typical

	5–9.9 g/d, 10–19.9 g/d, and ≥20 g/d), oral contraceptive use (ever/never), postmenopausal hormone use (ever/never), smoking status (nonsmoker, current smoker <25 cigarettes/d, current smoker ≥25 cigarettes/d), benign breast disease (yes/no), family history of breast cancer (yes/no), and physical activity (in quintiles of metabolic equivalents/wk [METs, the caloric need per kilogram body weight per hour activity, divided by the caloric need per kilogram per hour at rest]).	
Source of heterogeneity	Schernhammer et al (2001): NHS1 [15]: Reliable	Special characteristic
Selection	In 1976, a total of 121 701 female registered nurses 30–55 years of age and living in 11 large U.S. states were enrolled in the Nurses' Health Study. Since baseline, they have completed biennial-mailed questionnaires that comprise items about their health status, medical history, and known or suspected risk factors for cancer and heart disease. The questions include age, age at menarche, parity, age at first birth, weight, height, menopausal status, family history of breast cancer, and personal history of benign breast disease and cancer. Every 2 years, follow-up questionnaires have been sent to cohort members to update the information on potential risk factors and to identify newly diagnosed case subjects with cancer and other major medical events. In 1980, the questionnaire was expanded to include an assessment of diet and alcohol consumption. Follow-up data are available for more than 90% of the cohort.	Typical
Exposure	In 1988, the study participants were asked how many years in total they had worked rotating night shifts with at least three nights per month in addition to days or evenings in that month. Information on lifetime years worked on rotating night shift was gathered in eight prespecified categories: never, 1–2, 3–5, 6–9, 10–14, 15–19, 20–29, and 30 or more years. Of the 103 613 nurses who responded to the 1988 questionnaire, 85 197 answered the shiftwork question.	Typical
Outcome	Breast cancer cases were defined as having occurred during the period from June 1988 through May 1998. Nurses who reported the occurrence of breast cancer were asked for permission to review their medical records, and breast cancer was confirmed through review of these records. When medical records were unavailable, breast cancer cases were defined as probable and included in the analysis if they were corroborated	Typical

	by an interview or a letter from the subject. Approximately two thirds of the deaths among cohort members were reported to us by next of kin or the postal system in response to follow-up questionnaires. In addition, we searched the National Death Index to identify deaths among the nonrespondents to each 2-year questionnaire; the computerized National Death Index is a highly sensitive method for identifying deaths in this cohort. Data on mortality were more than 98% complete. For all deaths possibly attributable to breast cancer, we requested permission from family members (subject to state regulation) to review the medical records. Breast cancer was considered to be the cause of death if the medical records or autopsy report confirmed a fatal breast cancer, if the breast cancer was listed as the underlying cause of death without another, more plausible cause, and if the nurse was known (from hospital records, a family member's report, or another source) to have had breast cancer before death. In no case was the cause listed on the death certificate used as the sole criterion for death due to breast cancer.	
Confounding	Pooled logistic regression models were used to calculate RRs with adjustment for age, age at menarche (≤12, 13, and ≥14 years), age at menopause (≤43, 44–46, 47–49, 50–52, 53–55, 56–58, and >58 years), parity (nulliparous, 1–2, 3–4, and ≥5), age at first birth (<25, 25–29, and ≥30 years), weight change between age 18 years and menopause (<2, 2–9, 10–20, and ≥20 kg) for menopausal women only, body mass index (weight in kilograms divided by the square of the height in meters) at age 18 years in five categories (<21, 21–22.9, 23–24.9, 25–28.9, and ≥29 kg/m2), current alcohol consumption (nondrinkers <90 and ≥90 g/week), height in eight categories (≤150, 151–155, 156–160, 161– 165, 166–170, 171–175, 176–180, and >180 cm), oral contraceptive use (ever/never), use of postmenopausal hormones (never, past user <5 years, past user ≥5 years, current user <5 years, and current user ≥5 years), menopausal status, benign breast disease (yes/no), and family history of breast cancer (yes/no).	Typical

Case-control studies		
Source of heterogeneity	Bustamante et al. (2019) [2]: Unreliable	Special characteristic
Selection	We conducted a case-control study with incident cases of women histologically diagnosed with breast cancer at the Instituto de Seguridad Social del Estado de Mexico y Municipios (ISSEMyM) Cancer Center in the City of Toluca, State of Mexico. Controls were women who had received a clinical examination and mammography and who were free of the disease at the time of enrollment in the study. Controls were matched to the cases on age ± 5 years, place of residence, type of health insurance, and both cases and controls, were without a family history of breast cancer, to evaluate the environmental factors that may be associated in the pathological etiology of the disease.	Typical
Exposure	The research instrument used to conduct the personal interview was a health and environmental questionnaire that included general sociodemographic information (i.e., age, marital status, education, occupation, ever conducted night-work, number of years performing night-work and type of night-work performed), reproductive information and risk factors associated with breast cancer (i.e., age at onset of menarche, number of pregnancies, number of children, lactation history, height and weight to obtain body mass index (BMI), menopausal status, age at onset of menopause, smoking status and hormonal use).<br>In the present study, night-work was defined as "working from 9 pm to 7 am for at least one year". The study questionnaire included three questions related to nightwork: (1) Did you ever work a night shift that included any of the hours between 9 pm and 7 am? (2) For how long did you work at night?, and (3) What activities did you perform in your night-work?	Shift work was classified into only 2 categories: no night shift work and night shift work.
Outcome	For the recruitment and ascertainment of information of study participants, a research nurse visited the ISSEMyM Cancer Center every Tuesday on a weekly basis during the 10 month duration of the study. Women with a breast cancer diagnosis were given an appointment to conduct a personal interview.	Typical
Confounding	The multivariate model included age at menarche, menopausal status, BMI,	Typical

	lactation history, and smoking status adjusted by age, type of health insurance and residential status between cases and controls.	
Source of heterogeneity	Davis et al. (2001) [16]: Reliable	Special characteristic
Selection	Case patients were women aged 20–74 years with a new diagnosis of breast cancer (ICD-O site codes 174.0–174.9) from November 1992 through March 1995. Case patients were identified by the Cancer Surveillance System of the Fred Hutchinson Cancer Research Center, Seattle, WA, one of 10 population-based cancer registries funded by the National Cancer Institute, Bethesda, MD, as part of the Surveillance, Epidemiology, and End Results (SEER)1 Program. Of the 1039 eligible case patients identified, 813 (78%) agreed to participate. Control subjects were women between the ages of 20 and 74 years with no history of breast cancer, selected to be equal in number to the case patients and frequency matched according to 5-year age groups. Control subjects were identified by random-digit dialing with the use of a modification of the method described by Waksberg. Of the 20 148 phone numbers dialed, 95% were successfully resolved in terms of determining whether the phone was residential and whether an eligible woman lived in the household. Of 1053 eligible women selected as control subjects, 793 (75%) agreed to participate.	Typical
Exposure	Data collection took place from April 1993 through December 1995. An in-person interview was used to ascertain information on known or suspected risk factors for breast cancer, such as reproductive history, family history of breast cancer, and lifestyle factors (e.g., alcohol consumption and cigarette smoking). In addition, questions were included to obtain details on sleep patterns and habits in the 10 years before diagnosis (or reference date), lighting characteristics of the subject's bedroom for all homes occupied in the 10 years before diagnosis (or reference date), and lifetime occupational history.<br>The lifetime occupational history consisted of every job the subject held for 6 months or longer, including volunteer and military service. Details included beginning and end dates for each job, job title, full-time or part-time status, and	Rather simple

	the percentage of time worked at day, evening, or graveyard shift, using specific start and stop times in defining each shift.	
Outcome	Case patients were women aged 20–74 years with a new diagnosis of breast cancer (ICD-O site codes 174.0–174.9) from November 1992 through March 1995. Case patients were identified by the Cancer Surveillance System of the Fred Hutchinson Cancer Research Center, Seattle, WA, one of 10 population-based cancer registries funded by the National Cancer Institute, Bethesda, MD, as part of the Surveillance, Epidemiology, and End Results (SEER)1 Program. Of the 1039 eligible case patients identified, 813 (78%) agreed to participate.	Typical
Confounding	Logistic regression models conditional on 5-year age strata; odds ratios were adjusted for parity, family history of breast cancer (mother or sister), oral contraceptive use (ever), and recent (<5 years) discontinued use of hormone replacement therapy.	Rather simple
Source of heterogeneity	Fritschi et al. (2013) [17]: Unreliable	Special characteristic
Selection	Women aged between 18 and 80 years who had a first incident invasive breast cancer diagnosed between May 2009 and January 2011 were recruited for the Breast Cancer Employment and Environment Study (BCEES) (Girschik et al, 2012). We identified cases from the population-based Western Australian (WA) Cancer Registry, based on mandatory reporting of invasive cancer by pathology laboratories and other clinical sites. We excluded cases if their diagnosis was ductal carcinoma in situ or was not primary breast cancer, they previously had breast cancer, or their diagnosis date was 4213 days before the cancer registry report.<br>During the same time period, we randomly selected control women from the WA electoral roll, and frequency age-matched them to the expected distribution of cases. Enrollment to vote is compulsory for adult Australian citizens. Women with a previous diagnosis of invasive breast cancer were excluded. Further ineligibility criteria for both case and control participants included: incorrect address, deceased, too unwell to participate, inadequate English and not resident in WA	Typical

Exposure	For phase shift, we wished to identify those women whose central cycle had adapted to night shift but whose peripheral rhythms may not have. The central cycle is quicker to adjust when doing forward rotation rosters in which night shifts follow day shifts and then days off, than backward rotation in which day shifts follow night shifts (Haus and Smolensky, 2013). If the shift schedule had no pattern, or if there were 42 days off between finishing day and starting night shift, we conservatively assumed backward rotation, which required the woman to work a greater number of night shifts before being categorised as having high level exposure than a forward rotation. Exposure was classified as high if the job involved 44 nights forward rotation or 46 nights backward rotation, medium with 3–4 nights forward or 4–6 nights backward rotation, and low with 3 nights backward rotation. If night shift was done for more than a 4-week block, we downgraded the phase shift by one level on the assumption that peripheral rhythms would synchronise with central rhythms over this time.	Rather simple. The main focus is not night-shift work, but other variables including sleep patterns.
Outcome	Women aged between 18 and 80 years who had a first incident invasive breast cancer diagnosed between May 2009 and January 2011 were recruited for the Breast Cancer Employment and Environment Study (BCEES). We identified cases from the population-based Western Australian (WA) Cancer Registry, based on mandatory reporting of invasive cancer by pathology laboratories and other clinical sites. We excluded cases if their diagnosis was ductal carcinoma in situ or was not primary breast cancer, they previously had breast cancer, or their diagnosis date was 4213 days before the cancer registry report.	Typical
Confounding	Exposure in each time window was compared with the reference category of never exposed at that time. All analyses are adjusted for age group and all other time window variables in the table.	Severely simple. This simple confounder adjustment could cause a bias.
Source of heterogeneity	Grundy et al. (2013) [18]: Reliable	Special characteristic
Selection	Vancouver<br>Incident breast cancer cases were recruited from the BC Cancer Registry. Eligible cases were women, aged 20–80 years, with a diagnosis of either in situ or invasive	Typical

	breast cancer with no previous cancer history (except non-melanoma skin cancer) living in Vancouver, New Westminster, Richmond and Burnaby. Potential controls were cancer-free individuals from the Screening Mammography Program of BC recruited from breast screening clinics in the same geographic areas who consented to participate in research studies through routine screening mammography (available to women in BC aged 40–79 years). Controls were frequency-matched to cases by 5-year age group.<br>Kingston<br>Both, cases and controls, were recruited from the Hotel Dieu Breast Assessment Program in Kingston, Ontario. Women were eligible for the study if they were under 80 years of age, had no previous cancer history (except non-melanoma skin cancer), were not too ill to participate, and were not taking cancerpreventative drugs. Those consenting to be contacted were called by the study coordinator to confirm eligibility, and sent a package including study information, questionnaire and consent form. Cases were women with a subsequent diagnosis of either in situ or invasive breast cancer, while controls were women with either normal mammogram results or a diagnosis of benign breast disease, frequency matched by age as in Vancouver. Among those contacted, response rates were 59% among cases and 49% among controls, with a total of 131 cases and 164 controls included.	
Exposure	Night shift work exposure assessment<br>Lifetime exposure to night shift work was obtained from the occupational history. For any job held for at least 6 months, participants provided the industry and job title, start and end dates, average number of hours per week, percentage of time on day, evening and night shifts (as a continuous variable), as well as start and end times for each shift type. This information was used to categorise each job as either a ‘night shift’ or ‘non-night shift’ occupation. For the ʻmain’ analyses, night shift work jobs were classified as those where ≥50% of time was reported to have been spent on evening and/or night shifts, capturing both rotating and permanent night shift schedules. The total number of years spent employed in all ʻnight shift’ jobs	Typical

	was calculated for each individual. The influence of the proportion of evening and late night shifts required for a job to be considered 'night shift work' was investigated with definitions of 20, 40, 60, 80 and 100%, compared with the 50% used in the main analysis. An additional night shift work metric involving the use of start and end times of shifts, where those jobs including shifts that started or ended between 23:00 and 7:00 were considered night shift jobs, was also evaluated.	
Outcome	Vancouver<br>Incident breast cancer cases were recruited from the BC Cancer Registry. Eligible cases were women, aged 20–80 years, with a diagnosis of either in situ or invasive breast cancer with no previous cancer history (except non-melanoma skin cancer) living in Vancouver, New Westminster, Richmond and Burnaby. Potential controls were cancer-free individuals from the Screening Mammography Program of BC recruited from breast screening clinics in the same geographic areas who consented to participate in research studies through routine screening mammography (available to women in BC aged 40–79 years).<br>Kingston<br>Cases were women with a subsequent diagnosis of either in situ or invasive breast cancer, while controls were women with either normal mammogram results or a diagnosis of benign breast disease, frequency matched by age as in Vancouver.	Typical
Confounding	Multivariable unconditional logistic regression was used to calculate ORs and 95% CIs for the relationship between night shift work and breast cancer risk. Age (continuous) and centre (Vancouver/Kingston) were included in all models, and all other variables were selected using an all-possible-models manual backwards selection procedure,31 where potential confounders associated with breast cancer at $p<0.25$ were initially included in the modelling process, and only variables that changed the OR by >10% were retained in the final model. Potential confounders were ethnicity; household income; education; menopausal status; use of fertility drugs, oral contraceptives, non-steroidal anti-inflammatory drugs (NSAIDs), antidepressants and hormone replacement therapy (HRT); reproductive factors	Typical

	including ever having been pregnant, number of pregnancies, age at first birth, breastfeeding and age at first mammogram; family history of breast cancer among first-degree relatives; lifestyle factors, including smoking status, pack-years smoking and lifetime alcohol consumption; and body mass index (BMI).	
Source of heterogeneity	Hansen et al. (2001) [3]: Unreliable	Special characteristic
Selection	CASES<br>Altogether 7565 women with confirmed primary breast cancer, born in the period 1935–59 and 30–54 years of age at the time of diagnosis, were identified in the files of the Danish Cancer Registry, which contains information on diagnosis (ICD-7), names, sex, and unique personal identification number.<br>CONTROLS<br>One control subject per case was drawn at random from the files of the Central Population Registry, identified by name, sex, and personal identification number, and matched to the cases on year of birth and sex. Controls had to be alive without cancer and to have been an employee (member of the national pension fund) before the date of diagnosis of the corresponding case.	Typical
Exposure	WORK SCHEDULES<br>Information on occupational groups in which employees work predominantly at night was obtained from a nationwide interview-based survey on living and working environment conditions in 1976 among 2603 women.<br>Night work was most prevalent among unskilled, and relatively young female employees. Trades in which at least 40% of the female responders worked at night are listed in Table 1.	The exposure classification is all night work combined versus daytime work. A dose-response relationship could not be established from this study.
Outcome	CASES<br>Altogether 7565 women with confirmed primary breast cancer, born in the period 1935–59 and 30–54 years of age at the time of diagnosis, were identified in the files of the Danish Cancer Registry, which contains information on diagnosis (ICD-7), names, sex, and unique personal identification number.	Typical
Confounding	To adjust for socioeconomic status and reproductive factors (number of children,	Rather simple

	age at birth of first and last child), we estimated odds ratios (ORs) and 95% confidence intervals (CIs) by conditional logistic regression analysis using the statistical package EPICURE.<br>* Odds ratio adjusted for age, social class, age at birth of first child, age at birth of last child, and number of children.	
Source of heterogeneity	Menegaux et al. (2013) [19]: Reliable	Special characteristic
Selection	Eligible cases were women aged 25–75 years, newly diagnosed for breast cancer between 2005 and 2007 and residing in the French departements of ‘‘Cote d'Or'' or ‘‘Ille-et-Vilaine'' (administrative areas) at the time of diagnosis. Patients were recruited in the main cancer hospital of each area, as well as from smaller public and private hospitals that also recruited breast cancer patients, by specifically trained investigators. All breast cancer diagnoses were confirmed histologically. Among the 1,553 eligible cases identified during the study period, 163 refused to participate, 151 women could not be contacted and 7 died before the interview. Finally, 1,232 (79%) incident breast cancer cases were included in the study.<br>Controls were selected among general population women free of cancer and resident in the study areas at the time of the cases' diagnoses. For including controls, quotas by age were established as a preliminary to yield the control group similar to the case group in terms of age to achieve frequency-matching (10-year age group). Quotas by socio-economic status (SES) were also set a priori to control for potential selection bias arising from differential participation rates across SES categories.<br>The recruitment of controls was conducted as follows: phone numbers of private homes were selected at random from the telephone directory of each study area where unlisted numbers had first been recreated. A phone number was dialed up to 15 times at different times of the day and different days of the week until contact could be established with the residents. When a woman was living in the residence reached by phone, she was invited to participate to the study, as long as the predefined quota corresponding to her age group and socioeconomic status (SES)	Typical

	was not completed. When the quota was exceeded, the woman was excluded. To obtain the desired number of controls within the limits of age and SES categories, ~30,000 phone numbers were dialed for identifying 1,731 eligible controls. Among these, 1,317 (76%) accepted to participate to an in-person interview and were included in the study.	
Exposure	For each job held for at least 6 consecutive months, we obtained a description of the work tasks, workplaces, occupational exposures and work schedules. Women were asked whether they had worked for at least 1 hr between 11:00 pm and 5:00 am during all or part of each job. We characterized any night work period with the month and the year of beginning and ending, the usual number of nights per week, and the hour when the night shift started and ended. Any night work period was categorized as overnight (night shift of 6 consecutive work hours or more spanning the time period 11:00 pm–5:00 am), late evening (night shift ending between 11:00 pm and 3:00 am), or early morning (night shift starting between 3:00 and 5:00 am).	Typical
Outcome	Eligible cases were women aged 25–75 years, newly diagnosed for breast cancer between 2005 and 2007 and residing in the French departements of ‘‘Cote d'Or'' or ‘‘Ille-et-Vilaine'' (administrative areas) at the time of diagnosis. Patients were recruited in the main cancer hospital of each area, as well as from smaller public and private hospitals that also recruited breast cancer patients, by specifically trained investigators. All breast cancer diagnoses were confirmed histologically. Among the 1,553 eligible cases identified during the study period, 163 refused to participate, 151 women could not be contacted and 7 died before the interview. Finally, 1,232 (79%) incident breast cancer cases were included in the study	Typical
Confounding	Analyses were systematically adjusted for the original matching variables, i.e., age (5-year period) and study area, and for well-established risk factors for breast cancer categorized as follows: age at menarche (<12, 12:reference, 13, 14, 15 years and more), age at first full-term pregnancy (<22, 22–24:reference, 25–27, >27 years), parity categorized (nulliparous:reference, 1, 2, 3, 4þ children), current use of menopausal hormone therapy (Yes, No), family history of breast cancer in	Typical

	first-degree relatives (Yes, No), body mass index according to the WHO categories (<18.5, 18.5–24:reference, 25–30, >30), alcohol consumption (3 drinks/week:reference, 4–7 drinks/week, 8–14 drinks/week, >14 drinks/ week), and tobacco consumption (Never smokers: reference, former smokers, current smokers). Duration of night work was categorized into two groups according to the median value among controls (<4.5, ≥4.5 years or 4, >4 years for the analysis of night work before first full-term pregnancy). The average number of nights per week was categorized into two groups according to the median of the distribution among controls (<3, ≥3). Analyses were also conducted after stratification by age group (<55 years, ≥55 years) used as a proxy of menopausal status.	
Source of heterogeneity	O'Leary et al. (2006) [20]: Reliable	Special characteristic
Selection	The EBCLIS methods have been described in previous publications. Briefly, women eligible for EBCLIS were participants in the Long Island Breast Cancer Study Project (LIBCSP). LIBCSP cases were residents of Nassau and Suffolk counties on Long Island, New York, newly diagnosed with a first primary in situ or invasive breast cancer between August 1, 1996, and July 31, 1997. LIBCSP controls were also Long Island residents, frequency matched to the expected age distribution of cases by 5-year age groups. Controls less than 65 years of age were identified by random digit dialing; controls 65 years of age or older were selected from Health Care Financing Administration rosters.	Typical
Exposure	Employment and shift work. The LIBCSP interview included an occupational history of all jobs held for 6 months or longer since the age of 16 years, part-time or full-time, paid or unpaid, including military assignments; no information was collected on shift work. The EBCLIS interview confirmed the job title, location, and start and end dates for each job held during the 15-year period prior to the reference date (i.e., date of diagnosis for cases or identification for controls). The frequency (days per week, months, or years), duration (number of months or years), and type of shift work were ascertained for each of these jobs, and the interview asked separately about evening shifts (an evening shift could start in the	Typical

	afternoon and end as late as 2:00 a.m.) and overnight shifts (an overnight shift could start as early as 7:00 p.m. and continue until the following morning).<br>Shift-work exposure history. All analyses were based on data for 487 (85 percent) of 576 cases and 509 (87 percent) of 585 controls who worked during the 15 years prior to the reference date. Definitions of shift work were based on ever working in at least one job during the past 15 years that included 1) any shift work (i.e., any evening or overnight shift job); 2) any evening shift (i.e., including jobs with both evening and overnight shift work); 3) evening shifts only (i.e., excluding jobs with both evening and overnight shift work); 4) any overnight shift (i.e., including jobs with both overnight and evening shift work); and 5) overnight shifts only (i.e., excluding jobs with both overnight and evening shift work). The referent group for these analyses included women who had never held jobs involving shift work. Duration of evening shift work was categorized into groups according to the median number of years working in jobs with at least one evening shift per week, based on the distribution among the controls: 1) less than one evening shift per week (referent group), 2) one or more evening shift per week for fewer years than the median, and 3) one or more evening shift per week for the same or more years than the median. A similar categorization was used for duration of overnight shift work.	
Outcome	The EBCLIS methods have been described in previous publications (25–28). Briefly, women eligible for EBCLIS were participants in the Long Island Breast Cancer Study Project (LIBCSP). LIBCSP cases were residents of Nassau and Suffolk counties on Long Island, New York, newly diagnosed with a first primary in situ or invasive breast cancer between August 1, 1996, and July 31, 1997.	Typical
Confounding	For the models based on all women, we included the following covariates from the LIBCSP main questionnaire: age at reference date (continuous), parity (number of livebirths), education (less than high school or high school graduate as the referent versus some college, college graduate, or postcollege), first-degree family history of breast cancer (mother, sister, or daughter), and history of benign breast disease (yes/no). The premenopausal models included the same variables,	Typical

	with the addition of body mass index (weight in kilograms/height in meters squared, continuous) at the reference date. Additionally, the postmenopausal models included body mass index at age 20 years (kg/m2, continuous).	
Source of heterogeneity	Papantoniou et al. (2016) [21]: Reliable	Special characteristic
Selection	MCC-Spain Study is a population based multi-case–control study on frequent tumors in Spain that includes 23 hospitals in 12 different regions and assesses five types of cancer (breast, colorectal, prostate, stomach and chronic lymphocytic leukemia) using the same series of population controls for all cases.<br>We included 1708 incident breast cancer cases and 1778 population controls in ten participating centers (Asturias, Barcelona, Cantabria, Girona, Guipu´zcoa, Huelva, Leo´n, Madrid, Navarra and Valencia). Cases were women, aged 20–85 with a new histological confirmed diagnosis of breast cancer living in the catchment area of each hospital for at least 6 months. Control subjects are women with no history of breast cancer living in the same catchment area as cases. Controls were selected randomly from the rosters of General Practitioners at the Primary Health Centers (PHC) participating in the study that cover nearly all the population living in the corresponding area.	Typical
Exposure	Data was collected with face-to-face interviews performed by trained personnel. Lifetime occupational history was assessed for all jobs held for at least 1 year. For each job reported, detailed information was collected on job title, main activity or task performed, beginning and ending dates, shift type (day, night, rotating), exact time schedules, hours worked, and number of night shifts per month.	Typical
Outcome	We included 1708 incident breast cancer cases and 1778 population controls in ten participating centers (Asturias, Barcelona, Cantabria, Girona, Guipu´zcoa, Huelva, Leo´n, Madrid, Navarra and Valencia). Cases were women, aged 20–85 with a new histological confirmed diagnosis of breast cancer living in the catchment area of each hospital for at least 6 months	Typical
Confounding	OR adjusted for age, centre, educational level (less than primary, primary, high school, university), parity (nulliparous, 1–2, >3), menopausal status	Typical

	(premenopausal, postmenopausal), family history of breast cancer (yes/no), body mass index (<22.5, 22.5–25, 25–30, >30), smoking status (ever, never), oral contraceptive use (yes, no), leisure time physical activity (inactive, little active, moderately active, very active), alcohol consumption (quartiles), sleep duration (<7, 7–8,>8h)	
Source of heterogeneity	Pesch et al. (2010) [22]: Reliable	Special characteristic
Selection	GENICA was a population-based case–control study conducted among women from the Greater Region of Bonn, Germany, as previously described (17). In brief, 1143 incident breast cancer cases and 1155 population controls were enrolled between 2000 and 2004 with response rates of 88% and 67%, respectively. Cases were enrolled from the major hospitals of the region. Controls were ascertained as a random sample from the population registries of the study region and frequency matched to cases by age in 5-year classes. Inclusion criteria comprised age ≤80 years and Caucasian ethnicity.	Typical
Exposure	In addition, information on shiftwork status was sought (hereafter referred to as the “shift-status interview”). Of the 247 women reporting shift work, 223 (104 cases and 119 controls) could be reached for another telephone interview to collect detailed information on shift work for each occupational period (hereafter referred to as the “shift interview”). Comprehensive plausibility checks were applied to assess night work and lifetime exposure to night shifts. Night work was defined as working the fulltime period between 24.00–05.00 hours according to the International Labor Organization (ILO) convention on night work. An almost equal number of 56 cases and 57 controls had ever worked in night shift for ≥1 year. Shift work and other information were truncated at the date of the core interview.	Typical
Outcome	Incident cases were women with histopathologically confirmed breast cancer diagnosed within six months before enrolment.	Typical
Confounding	The set of potential confounders comprised menopausal status (pre- or postmenopausal), education, breast cancer in mother or sister, parity (nulliparous,	A relatively small number of confounders were included in the final model. However, the authors

	1–2, ≥3 children), age at first birth (nulliparous, <25, 25–29, ≥30 years), duration of oral contraceptive and hormone therapy use (never, >0–9, ≥10 years), body-mass index (<22.5, 22.5–<25, 25–<30, and ≥30 kg/m2), smoking (current, former, never), number of mammograms until two years before interview, and lifetime breastfeeding in months. Women were considered pre-menopausal if they reported bleedings in the year of interview and no bilateral oophorectomy. They were categorized as "low educated" if they had no occupational qualification and ≤8 years at school and "highly educated" if they had a university degree; otherwise education level was classified as "medium".<br>Each variable was added one at a time in order to assess whether it would modify the association between night work and breast cancer. None of the potential confounders changed the risk estimate of night or shift work by more than 10%. In the final model, a variable was considered a potential confounder if the respective risk estimate reached a significance level of 15%. The final models included family history of breast cancer, hormone therapy use, and number of mammograms.	carefully examined other confounders before excluding them from the final model.
Source of heterogeneity	Pham et al. (2019) [23]: Reliable	Special characteristic
Selection	Women aged 20 years old or more who visited the Breast Cancer Center or Health Examination Center at the National Cancer Center in Korea from 2012 February to 2018 January were enrolled. Eligible cases of incident breast cancer were histologically confirmed by pathologists and breast cancer surgeons. Controls were selected from among women who visited the centers for health check-ups between 2012 and 2016.<br>Controls were confirmed as being free-of-cancer based on medical record review, results of health checkup, and answers of questionnaires regarding past medical history. If they had any type of cancers, they were excluded from the analysis.	Typical
Exposure	NSW was defined as ever having worked in night shifts regularly between 9:00 pm and 8:00 am for at least 2 months in their lifetime. In addition, age at starting and ending NSW, clock-time of starting and finishing night shifts, the number of	Typical

	working days per week, the average length of shift cycles, and years on night schedule were obtained using a standardized questionnaire. NSW exposure was classified as having ever or never engaged in night work, in addition to the length of exposure and the shift schedule. Shift schedule was detailed for age at starting to work night shifts, number of days per week for NSW exposure, clock time at starting night shifts, and lifetime duration of night work as recommended by the IARC working group.26 The duration of NSW involvement was classified into three levels: 10 years and under, longer than 10 years. To integrate the shift duration and intensity, the lifetime cumulative hours of night work were computed by multiplying the number of years with the number of days per week with NSW, and hours of working per NSW.	
Outcome	During the enrollment period, a total of 2058 female patients with breast cancer and 1938 female controls with NSW information were recruited. The cases and controls were matched by 10-year age groups. For breast cancer patients, pathology reports were reviewed to access information about the hormone receptor status of breast cancer tissue including estrogen receptors (ER), progesterone receptors (PR), and human epidermal growth factor receptor 2 (HER2). After checking medical records of enrolled patients, we classified breast cancer into four subtypes based on the receptor status: luminal A (ER and/or PR positive and HER2 negative), luminal B (ER and/or PR positive and HER2 positive), HER2enriched (ER and PR negative and HER2 positive), and triple-negative (all receptors negative).	Typical
Confounding	The fully adjusted ORs incorporated all established risk factors including age, number of pregnancies, age at birth of first child, educational levels, BMI, age at menarche, tobacco smoking, use of female hormone treatment, and family history of breast cancer to yield a fully adjusted result. This was performed by multivariate conditional logistic regression using never worked in night shift as the reference.	Typical
Source of heterogeneity	Wang et al. (2015) [4]: Unreliable	Special characteristic
Selection	Female patients with recent histologically diagnosed primary breast cancer	Typical

	between July 2010 and March 2012 in the First- and Second-Affiliated Hospitals and Sun Yat-sen University Cancer Center, Guangzhou, China, were consecutively included in this study. Incident cases were immediately interviewed after admission and before treatment. Women with metastasized breast cancer or previous history of any cancers were excluded. A total of 712 eligible breast cancer patients completed face-to-face interviews with response rates ranging from 75% to 85% depending on different hospitals. Of the 712 cases, 661 (92.8%) cases answered the question of night-shift work, 654 (91.9%) cases answered the question of 24-h sleep duration, and 658 (92.4%) cases answered the question about daytime napping.<br>Controls were recruited from women who attended a health checkup in the same hospitals as the breast cancer patients during the same period, and they were frequency-matched to patients by 5-year age groups. Women with major chronic diseases or who self-reported a history of cancer were excluded. Of the eligible controls, 742 (78.2%) completed in-person interviews. The response rates for the sleep-related variables were comparable to those among the breast cancer cases: 714 (96.2%) for night-shift work, 667 (89.9%) for 24-h sleep duration, and 674 (90.8%) for daytime napping.	
Exposure	For sleep-related variables, participants were asked about the following questions: (1) In your lifetime (adulthood), have you ever engaged in night-shift work or other activities at night, at least once a week and last for six months or more (yes vs. no)? Night-shift work or other activities at night was defined as being awake or working between midnight and 6:00 AM.	In addition to night shift work, 24-h sleep duration and daytime napping were included as a main exposure variable. For night shift work, the exposure assessment was classified into only 2 categories: ever and never night shift worker. This causes a serious limitation in estimating a dose-response relationship.
Outcome	Female patients with recent histologically diagnosed primary breast cancer between July 2010 and March 2012 in the First- and Second-Affiliated Hospitals and Sun Yat-sen University Cancer Center, Guangzhou, China, were consecutively included in this study. Incident cases were immediately interviewed after admission and before treatment. Women with metastasized breast cancer or	Typical

	previous history of any cancers were excluded. A total of 712 eligible breast cancer patients completed face-to-face interviews with response rates ranging from 75% to 85% depending on different hospitals. Of the 712 cases, 661 (92.8%) cases answered the question of night-shift work, 654 (91.9%) cases answered the question of 24-h sleep duration, and 658 (92.4%) cases answered the question about daytime napping.	
Confounding	Multivariate logistic regression models were used to assess the associations between sleep-related variables and breast cancer risk. The multivariate models were adjusted for age (continuous) and potential risk factors of breast cancer, including education (college or above, senior high school vs. junior high school), body mass index (BMI; ≥25.0, 23.0–24.9 vs. < 23.0), age at menarche (>12 vs. ≤ 12), parity (≥1 vs. 0), menopausal status (postmenopausal vs. premenopausal, postmenopausal was defined as the absence of menstrual periods for 12 months), breast-feeding (ever vs. never), physical activity (≥18,3to <18, vs. < 3 Met-h/week/year), and a family history of breast cancer (yes vs. no). Sleep-related variables were also included for mutual adjustment.	Typical
Source of heterogeneity	Yang et al. (2019) [5]: Unreliable	Special characteristic
Selection	The JBCS (Yang et al., 2017) is an ongoing populationbased case–control study to assess the risk of female BC associated with sleep factors and other potential risk factors in Jiujiang City, China, and was initiated in 2013. The source population for the JBCS included female permanent residents aged 18–74 years in two districts (i.e. Xunyang and Lushan) in Jiujiang. The incident BC cases were identified through the local cancer registries in the two districts from January 2013 to December 2016. All possible cancer cases were verified through home visits and further review of medical charts by clinical and/ or pathological experts. To be eligible for participation, case patients had to be: (a) newly diagnosed with a histologically confirmed invasive BC; (b) a permanent resident in one of the geographically defined study regions; and (c) 18–74 years old. Of the 432 eligible BC cases identified, 29 refused and two had died. Thus, a total of 401 (93%)	Typical

	female BC cases were included in the current study.<br>We matched one population control for each case by sex, year of birth (within 1 year), and region of residence (according to the patient's house number) during the year when the case was diagnosed with cancer. For example, a 45-year-old woman with the house number of 6707 was newly diagnosed with BC and was enrolled in the study. Then, her female neighbor with the house number of 6709 was selected as her individually matched control if she (a) was aged 44–46 years; (b) was free of BC; and (c) agreed to take part in the study. Otherwise, the female neighbor with the house number of 6711 was asked to confirm whether she fulfilled the above selection criteria, and so forth, till the appropriate control was found.	
Exposure	For night/shift work, participants were asked whether they had ever had night/shift work (yes/ no) and, if yes, the frequency (per week), the amount in hours per day, and duration (years) of night/shift work were recorded. For habitual nap time, answer categories included 0, 0–1, 1–2, and greater than 2 h per day. In addition, patients with BC were further asked whether their sleep habits including sleep duration or subjective sleep quality were largely changed after they developed the disease.	For night shift work, the exposure assessment was classified into only 2 categories: ever and never night shift worker. This causes a serious limitation in estimating a dose-response relationship.
Outcome	The incident BC cases were identified through the local cancer registries in the two districts from January 2013 to December 2016. All possible cancer cases were verified through home visits and further review of medical charts by clinical and/ or pathological experts. To be eligible for participation, case patients had to be: (a) newly diagnosed with a histologically confirmed invasive BC; (b) a permanent resident in one of the geographically defined study regions; and (c) 18–74 years old. Of the 432 eligible BC cases identified, 29 refused and two had died. Thus, a total of 401 (93%) female BC cases were included in the current study.	Typical
Confounding	Adjusted for age (<45, 45–60, ≥60 years), education (≤elementary school, middle school, high school, ≥ prof/tech/college), family income (low, middle, and high), occupation (housewife, professional, clerical, and manual worker), menopausal status (yes/no), number of live births (0, 1, 2, 3, ≥ 4), use of menopausal hormones	Typical

	(yes/no), age at menarche (<12, 12–13, ≥13 years), age at first birth (<20, 20–25, 25–30, ≥30 years), marital status (married, never married, widowed, divorced), family history of breast cancer (yes/no), smoking (yes/no), alcohol drinking (yes/no), fruit and vegetable consumption (g/day), regular physical activity (yes/no), body mass index (<18.5, 18.5–24.0, 24.0–28.0, ≥28 kg/ m2), and adjusted mutually for other sleep variables including sleep duration (< 6, 6–7, 7–8, >8 h), sleep quality (very good, fairly good, fairly poor, very poor), light exposure at night (level 1, level 2, level 3, level 4), night/shift work (yes/no), and sleep medication use (yes/no).	
Source of heterogeneity	Hansen et al. (2012a): Danish military [24]: Reliable	Special characteristic
Selection	All 329 cases of primary breast cancer (ICD-7 170) in 1990–2003 were documented from the files of the nationwide Danish Cancer Registry by use of the Central Person Registry number. The Cancer Registry, which was initiated in 1942, is regarded as virtually complete and contains diagnoses classified by an extended Danish version of the International Classification of Diseases, revision 7, and date of diagnosis. In total, 218 women who had had breast cancer (66%) were still alive and living in Denmark at the time of interview (between March 2005 and December 2006). We received mandatory permission from the treating hospitals to contact 210 (96%) of these live cases. We chose 899 live potential female controls at random from the cohort by incidence density sampling and approximately frequency matched (1:4) them with cases on the distribution of 1-year groups of birth years. The current address of each potential participant was retrieved from the Central Person Register, which is updated daily.	Typical
Exposure	Night shift work was defined as working for at least 1 year during hours beginning after 17:00 and ending before 9:00, not including overtime. This includes exposure to artificial light after midnight, when melatonin normally peaks in healthy people.20 In this definition, permanent and rotating night shifts were assessed as one entity. We recorded first calendar year, total duration (1-2, 3-5, 6-9, 10-15, 15-19, 20-29 and ≥30 years) and the average number (continuous) of such shifts per	Typical

	week in periods with night work. We did not separate out night shift work with the military from that outside. Cumulative exposure (ie, lifetime number of night shifts) was calculated as the average number of night shifts per week multiplied by the midpoint of the upper and lower boundaries of the duration of shift work categorised in the questionnaire. Tertiles for exposed controls were used as cut-off points in the analyses.	
Outcome	All 329 cases of primary breast cancer (ICD-7 170) in 1990–2003 were documented from the files of the nationwide Danish Cancer Registry by use of the Central Person Registry number. The Cancer Registry, which was initiated in 1942, is regarded as virtually complete and contains diagnoses classified by an extended Danish version of the International Classification of Diseases, revision 7, and date of diagnosis. In total, 218 women who had had breast cancer (66%) were still alive and living in Denmark at the time of interview (between March 2005 and December 2006). We received mandatory permission from the treating hospitals to contact 210 (96%) of these live cases.	Typical
Confounding	Potential confounders were evaluated in univariate analyses based on Student t test for continuous variables and c2test for categorical variables: length of education (7, 8–9, 10, >10 years), body mass index (<25, ≥25 kg/m2), alcohol drinking (cumulative, ever, never), menopausal status (pre, post), use of hormone replacement therapy (never, 1–5, ≥6 years), use of contraceptives (never, 1–9, ≥10 years), occupational exposure to radar or EMF (yes/no), occupational physical activity (no, light, heavy), satisfactory influence on job (yes/no), too high a workload and work pace (yes/no), age at menarche (<11, 11–14, ≥14 years), age at menopause (<45, 45–49, 50–54, ≥55 years), number of childbirths (0, 1–2, ≥3), tobacco smoking (0, 1–10, ≥10 years, never, current, previous, continuous) and occasional sun exposure (never/rare, at least once weekly/always). In the final models, we included, in addition to night shift work variables, the following variables with univariate p values ≤0.15: hormone replacement therapy (0.003), number of childbirths (0.15), age at menarche (0.02), length of education (0.01), occasional sun-bathing (0.01) and tobacco smoking status (0.15). Inclusion of the	Typical

	other variables did not change the risk estimates substantially (table 1)<br>(table footnote) Adjusted for age, hormone replacement therapy (0, 1e5, $6 years), number of childbirths (0, 1–2, ≥3), age at menarche (<11, 11–14, ≥14 years), years of education (7, 8–9, 10, ≥11), occasional sunbathing frequency (never/rare vs always/more than weekly) and tobacco smoking status (never, current, former).	
Source of heterogeneity	Lie et al. (2006) [25]: Reliable	Special characteristic
Selection	Cohort information<br>A cohort of 44,835 women who graduated from a three year nursing school between 1914 and 1980, and were alive 01.01.1953 or born later, was established, based on information from the Norwegian Board of Health's registry of nurses. This registry was established in 1949, and included all nurses who graduated from a Norwegian nursing school and were alive in 1949, or born later. It also included nurses with graduation from other countries, who had obtained a Norwegian authorization. Cancer cases were identified by linkage to The Cancer Registry of Norway, which has virtually complete records of individual cases of cancer in the Norwegian population since 1 January 1953. The coding of cancers is based on a modified version of the 7th revision of the International Classification of Diseases (ICD-7)<br>Nested case–control study<br>A nested case–control study was carried out within the cohort. Since data on fertility was available only from 1960, it was decided to include cancer cases diagnosed from this year. Inclusion of new breast cancer cases ended in 1982, after which year data on individual work histories were no longer complete. A total of 537 first occurrence breast cancer cases were identified by linkage to the Cancer Registry. The linkage was based on the personal identification number introduced to all citizens alive in 1960, or born later. All dates of death and emigration were obtained from Statistics Norway by the same linkage procedure.<br>For each case four controls, alive and without breast cancer at the time of diagnosis of the case, were randomly drawn from the nurse registry according to the	Typical

	incidence density sampling method. The controls were matched by year of birth of the case (–1 year) and were required to have graduated (or started in the first job) the year the case was diagnosed at the latest.	
Exposure	Exposure<br>From the time a three-year nursing education was established in Norway (around 1900) and several decades onwards, almost all nurses who were employed at infirmaries worked rotating night shifts. The burden of night work has varied between hospitals and departments during the years, as has the use of permanent night workers, which became more common from the middle of last century. Nurses with rotating night shifts have usually worked at least three nights per month.<br>The reconstruction of total work history as a nurse and number of years with night work was based on individual information from two sources, the Norwegian Board of Health's registry of nurses, from which the cohort was formed, and census data from 1960, 1970, and 1980. Number of working years was accumulated from first year of employment until termination of the last employment, date of diagnosis of the case, or the 67th birthday, whichever came first. Work history from the nurse registry was self-reported, and for each employment as a nurse included beginning and end dates, work place, and to some extent, department/ward. Until 1960 the registry was updated yearly. The last regular update was performed in 1968, and thereafter only sporadically. Data about occupation and industry at the time of the censuses in 1960, 1970, and 1980 was obtained by individual record linkage to the censuses at Statistics Norway. In the present study the median duration of employment in one job was four years. A period of maximum four years was therefore imputed to total work time around 1960, 1970, or 1980, if no other work history was available for these years, and the census' work code was 'nursing' or 'nursing and other care work' or the industry code was 'health work'.	Typical
Outcome	A total of 537 first occurrence breast cancer cases were identified by linkage to the Cancer Registry. The linkage was based on the personal identification number	Typical

	introduced to all citizens alive in 1960, or born later. All dates of death and emigration were obtained from Statistics Norway by the same linkage procedure.	
Confounding	* OR adjusted for total employment time as a nurse (0–2, 3–14, 15–29, ≥30 years) and parity (0, 1–2, 3 or more children)<br>Fertility factors may be associated with occupational history, and were thus considered as possible confounders. Information on age at birth of first child and number of children was obtained from Statistics Norway, for all cases and controls. To control for other possible occupational hazards than night work adjustments were made for total employment time, as no specific data on individual exposures were available.	A relatively small number of confounders were adjusted.
Source of heterogeneity	Szkiela et al. (2021) [26]: Reliable	Special characteristic
Selection	The case–control study was conducted in 2015–2019 in the Lodz region. The case group included 494 women over 35 years old, who had been diagnosed with malignant breast cancer and had a tumor resection or mastectomy. The control group included 515 women without breast cancer. The women from the case group were patients of the Oncological Surgery Department and the Second Department of Oncological Surgery, Oncological Surgery Clinics of the Provincial Specialist Hospital M. Kopernik in Lodz; the surgery department of Poddebice Health Center; the Provincial Specialist Hospital M. Skłodowska-Curie in Zgierz. The anthropometric data of the research participants were assessed using a self-assessment questionnaire.	Typical
Exposure	Respondents were asked whether they ever worked for at least 6 months. We were interested in all types of employment: full-time or part-time work, self-employment, work in a family business, and work in the army or on a farm. If respondents ever worked for at least 6 months, they were asked if they ever worked a night shift, with the possible answer options being “Yes” and “No.” Respondents working night shifts were asked whether they had worked three nights or less on a specific shift or more than three consecutive night shifts, and then they were transferred to the next shift. We also asked about the order of the	Typical

	shifts. If the order of shifts was I (morning)→II (afternoon)→III (night), we named it the "forward rotation." If the order of shifts was III→II→I, we named it the "backward rotation." The respondents were also asked how many times a month they worked at night. Finally, we also asked about the starting point for shift work, with possible answers being "Within 12 months before breast cancer," "1–5 years before breast cancer," "5–10 years before breast cancer," "Over 10 years before breast cancer." Finally, from the group of night shift employees, we derived four variables measuring the intensity of such work:<br>• 3 days or less/more than 3 days of consecutive night shifts (one after another);<br>• Forward/backward rotation of shift work;<br>• Less/more than 10 years of night work before illness developed;<br>• Number of night work years.	
Outcome	The outcome variable was breast cancer. The criteria for including women in the case group were histopathologically confirmed breast cancer or/and mastectomy and no history of other cancers. The criterion for including women in the control group was no history of breast cancer.	Typical
Confounding	Each of the A–G columns of Table 3 shows statistically significant ORs from separate logit regressions with the same set of the confounding factors: BMI > 25, breastfeeding < 6 months, menstruation age: 10–12, menopause age: 55+, living in the countryside, widow, smoking, and no pregnancies (for each variable, the reference group contained all other respondents who did not meet the given condition), as well as different night work intensity: (A) any night shift work, (B) working nights more than ten years before illness, (C) forward rotation of night work, (D) more than three consecutive night shifts, (E) more than 10 years of working nights, (F) trend of years of working nights, and (G) more than 10 years of working nights and more than three consecutive night shifts. The purpose of the analysis from Table 3 was to show how the intensity of shift work, defined using A–G, affected disease with the control of the same set of confounding factors.	Typical
Source of heterogeneity	Hansen et al. (2012b): Danish nurse [27]: Reliable	Special characteristic

Selection	An interview-based case–control study was nested within an established national cohort of 91,140 female members of the Danish Nurses Association, covering over 95% of nurses in Denmark. Information on all nurses is computerised on the basis of their unique personal identification number, which includes the date of birth and sex, and is assigned to all residents of Denmark by the Central Person Register. This number can be used to obtain updated computerised information on e.g. vital status, date of death, immigration and current address. This number is also the key identifier for cancer diagnoses in the nationwide Danish Cancer Registry, initiated in 1942.	Typical
Exposure	For each job held for at least 1 year, we assessed information on year or age of start and end, average number of hours worked per week, time of starting and ending on each shift in a normal week (excluding irregular overtime work). If the woman had worked in rotating shifts, we classified the job according to the most frequent occurring rotating shift system in Danish nurses: day–evening, day–night, day–evening night or others. For most of the nurses, day work was from 6 or 7 to 15 or 16, evening work from 15 or 16 to 23 or 24 and night work from 23 or 24 to 7 or 8. We used two series of partly overlapping variables to characterise the shift-work in each job held: type of shift (permanent day, rotating day–evening, day–night, day–evening– night, other rotating and permanent night), with calculated lifetime duration of each shift system weighted by hours worked per week; and number of shifts after midnight per month ('graveyard shifts', beginning after 19 and ending before 9), with calculated lifetime cumulative numbers of such shifts based on the duration and number per month, including tertiles based on the distribution in exposed controls. If subjects reported years of after midnight shifts, but were unable to provide numbers of such shifts per month (4.7% of cases and 4.8% of controls), we imputed this information by the average number for cases and controls, respectively. Overall, this changed final results only marginally.	Typical
Outcome	Information on all nurses is computerised on the basis of their unique personal identification number, which includes the date of birth and sex, and is assigned to	Typical

	all residents of Denmark by the Central Person Register. This number can be used to obtain updated computerised information on e.g. vital status, date of death, immigration and current address. This number is also the key identifier for cancer diagnoses in the nationwide Danish Cancer Registry, initiated in 1942.	
Confounding	The final models included, in addition to the shift-work variables, breast cancer in mother or sister, use of hormone replacement therapy (never, <5, P5 years), lifetime cumulative months of breastfeeding, , age at first child birth (nulliparous, <25, 25–29, 25–30, P35 years), age at menarche (continuous), menstrual regularity, weight fluctuations and menstrual status.	Typical

Supplementary material G. Representative RRs and ORs from cohort and case-control studies, respectively (included in each forest plot)

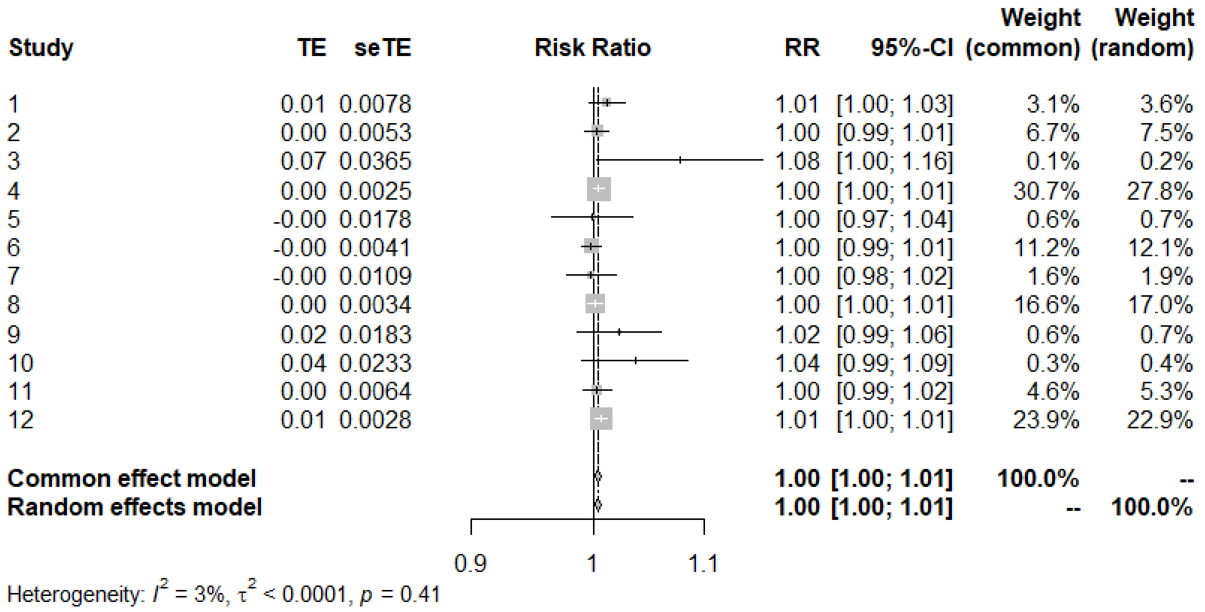

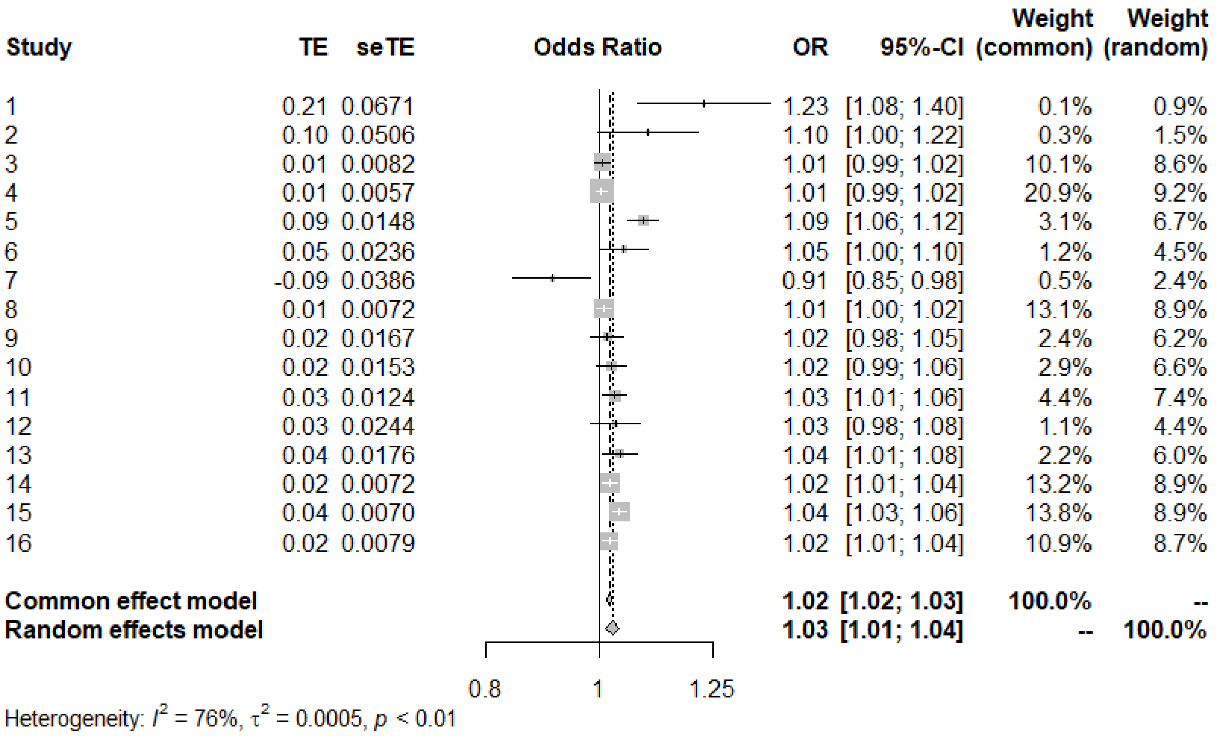

Supplementary material H. Results of dose-response meta-analyses (all studies) and dose-response meta-analysis plot for cohort and case-control studies (all studies)

study type	years of night shift work			
	1 year	10 years	20 years	30 years
	the pooled RR and pooled OR for female breast cancer (95% CI)			
cohort studies	1.0042 (1.0015-1.0069)	1.0428 (1.0151-1.0712)	1.0873 (1.0303-1.1475)	1.1338 (1.0459-1.2292)
case-control studies	1.0279 (1.0143-1.0417)	1.3171 (1.1530-1.5044)	1.7346 (1.3294-2.2633)	2.2846 (1.5328-3.4051)

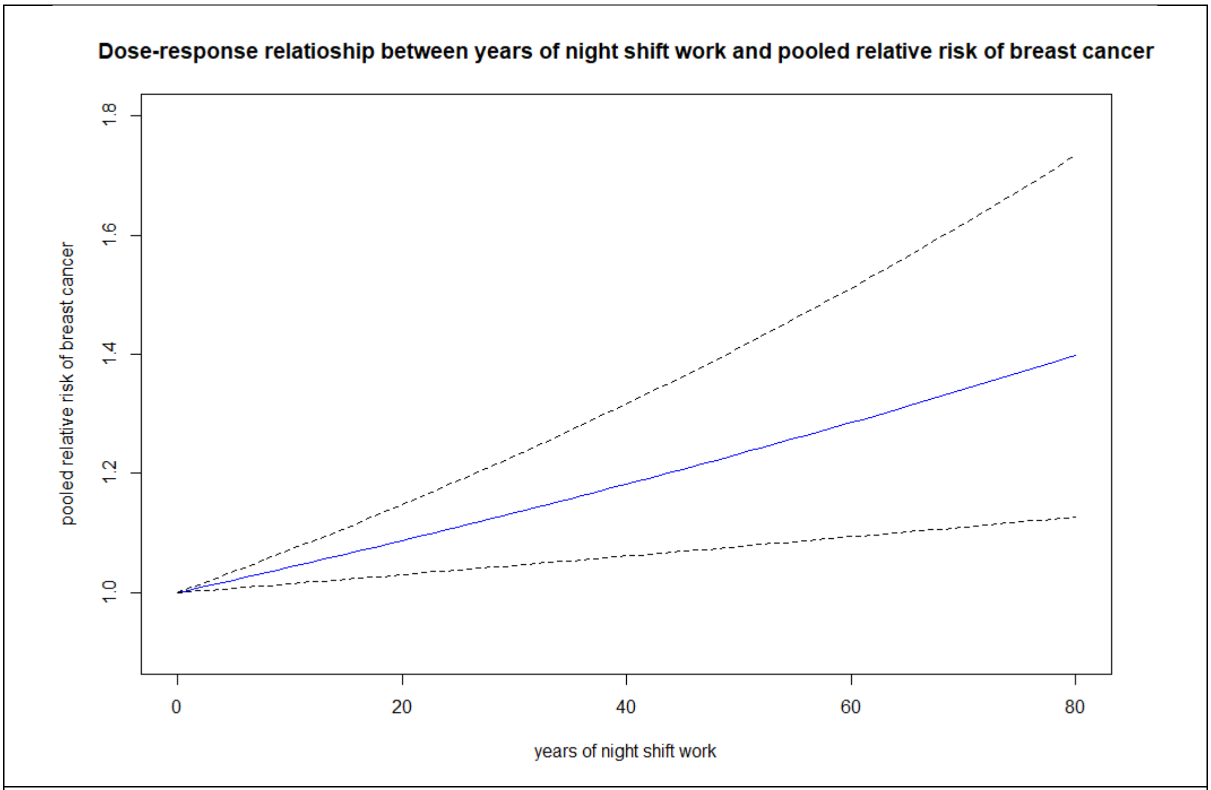
Dose-response relatioship between years of night shift work and pooled relative risk of breast cancer
pooled relative risk of breast cancer
1.0
1.2
1.4
1.6
1.8
0
20
40
60
80
years of night shift work

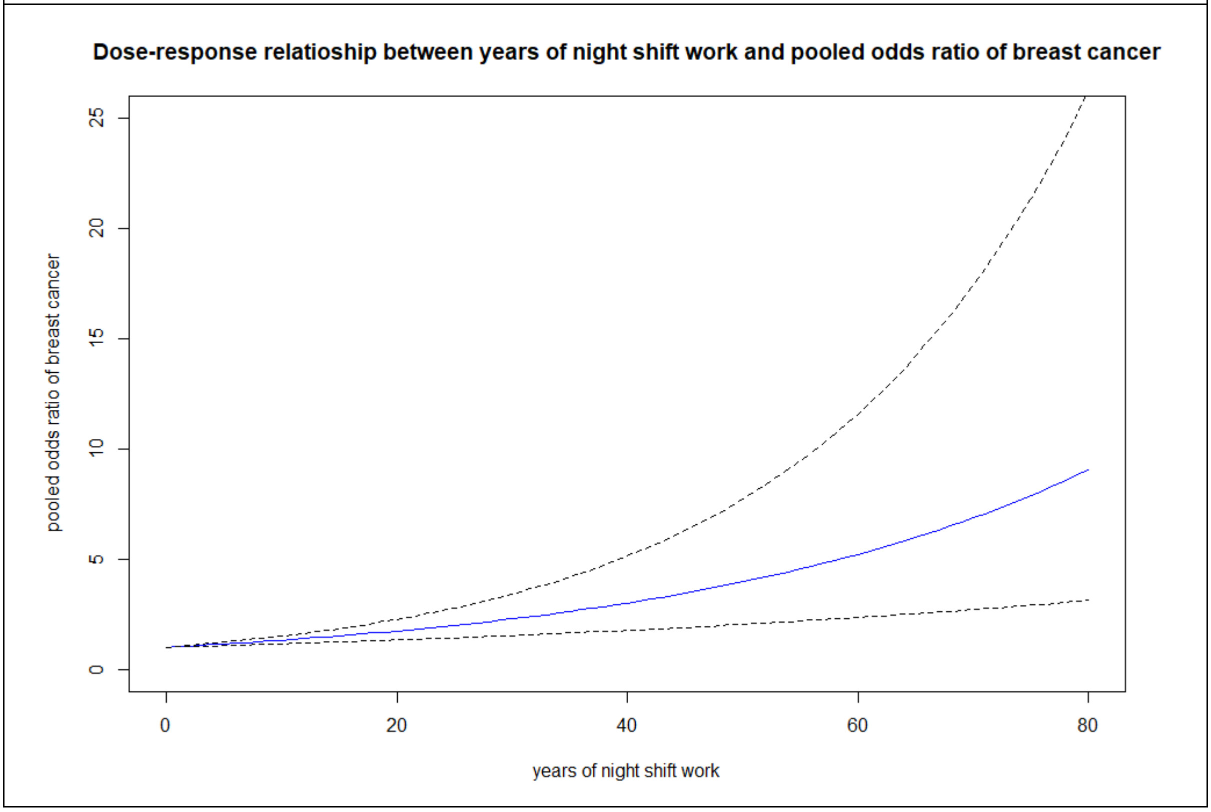
Dose-response relatioship between years of night shift work and pooled odds ratio of breast cancer
pooled odds ratio of breast cancer
0
5
10
15
20
25
0
20
40
60
80
years of night shift work

SUPPLEMENTARY TEXTS

Supplementary texts A. Potential biological mechanistic connections between night shift work and breast cancer

Haus and Smolensky summarized four biological mechanisms of carcinogenesis in night shift workers [28]. (i) Light at night induces circadian disruption and phase alteration that induces a variety of internal/cellular environments favoring carcinogenesis, including epigenetic modification. (ii) Night shift work induces faulty cell division and DNA repair with malignant potential. (iii) Night shift work induces melatonin suppression. The function of melatonin includes neutralizing and removing free radicals, inhibiting the uptake of free acids (necessary for the local formation of mitogens) in potential and growing tumor cells, and controlling normal estrogen metabolism. The inhibition of these functions makes the risk of breast cancer increase. (iv) Sleep deprivation in night shift workers induces compromised immune surveillance and metabolic disarrangement. Metabolic disarrangement is associated with a tendency for obesity and a low-grade inflammatory state with the generation of reactive oxygen species. These compromised immune surveillance and increased reactive oxygen species elevate the risk of carcinogenesis. NTP concluded that there is sufficient evidence of carcinogenicity between night shift work (or light at night) exposure and breast cancer, based on a collective body of cancer epidemiology and mechanistic studies in humans. They summarized that several animal cancer studies measured biological effects that are associated with circadian disruption or characteristics of carcinogens, and these studies provided a link between night shift work (or light at night) exposure, intermediate biological effects, and cancer incidence [29].

Supplementary texts B. Association between light-at-night exposure and breast cancer incidence

In consideration of the biological mechanism between night shift work exposure and breast cancer incidence, light at night exposure can be considered as a similar health hazard. In 2021, Urbano et al. published a systematic review and meta-analysis article on the association between light exposure at night and breast cancer [30]. In this study, the pooled RR for cohort studies was 1.10 (95% CI 1.06-1.15), and the pooled OR for case-control studies was 1.14 (95% CI 0.98-1.34). In addition, premenopausal women showed a relatively higher RR than postmenopausal women (RR of 1.16, 95% CI 1.04-1.28 for premenopausal women vs. RR of 1.07, 95% CI 1.02-1.13 for postmenopausal women).

Numerous studies reported a decreased incidence of breast cancer in women with total visual blindness. Flynn-Evans et al. reported that women with no perception of light had a lower prevalence of breast cancer than women with light perception (OR of 0.43, 95% CI 0.21-0.85) [31]. Pukkala et al. reported that breast cancer risk in females decreased by the degree of visual impairment and that a similar but less consistent trend was observed for prostate cancer in males [32]. Kliukiene et al. reported that the risk of breast cancer was lower (but statistically insignificant because of a few numbers of cases) in totally blind women (SIR of 0.64, 95% CI 0.21-1.49) and women who became blind before the age of 65 (SIR of 0.51, 95% CI 0.11-1.49) [33]. Verkasalo et al. reported that the SIR of breast cancer was reduced along with the increasing degree of visual impairment [34]. For the point estimate of SIR, the test for trend showed a p-value of 0.036. In 1998, Feychting et al. reported that totally blind people had a lower incidence of all cancers combined (standardized incidence ratio (SIR) of 0.69, 95% CI 0.59-0.82) [35]. The risk reduction was prominent in hormone-dependent tumors, including breast and prostate cancer.

Supplementary texts C. Effect modification by hormone receptor status

Hong et al. reported that night shift work increased the risk of breast cancer ('pooled HR' of 1.20, 95% CI 1.10-1.31) [36]. In particular, estrogen receptor (ER) positive, progesterone receptor (PR) positive, and human epidermal growth factor receptor 2 (HER2) positive breast cancer showed the pooled HR of 1.35 (95% CI 1.19-1.53), 1.30 (95% CI 1.09-1.54), and 1.42 (95% CI 1.17-1.72), respectively. However, the pooled HR for ER/PR negative/negative and HER2 negative breast cancer showed 0.98 (95% CI 0.80-1.20) and 1.10 (95% CI 0.83-1.46), respectively. Another study reported that night work for ≥20 years was associated with a statistically significant elevated risk of ER-negative breast cancer (OR 4.73, 95% CI 1.22-18.36) based on a population-based case-control study named Gene-Environment Interaction and Breast Cancer (GENICA) study [37]. Even though these studies showed different results with each other, the segregation of breast cancer according to receptor status (ER+/-, PR+/-, and HER2+/-) is worth considering in future studies.

REFERENCES

1. Knutsson A, Alfredsson L, Karlsson B, Åkerstedt T, Fransson EI, Westerholm P, Westerlund H: **Breast cancer among shift workers: results of the WOLF longitudinal cohort study**. *Scand J Work Environ Health* 2013, **39**(2):170-177.
2. Bustamante-Montes LP, Flores-Meza B, Hernández-Valero MA, Cárdenas-López A, Dolores-Velázquez R, Borja-Bustamante P, Borja-Aburto VH: **Night Shift Work and Risk of Breast Cancer in Women**. *Archives of Medical Research* 2019, **50**(6):393-399.
3. Hansen J: **Increased Breast Cancer Risk among Women Who Work Predominantly at Night**. *Epidemiology* 2001, **12**(1).
4. Wang P, Ren F-M, Lin Y, Su F-X, Jia W-H, Su X-F, Tang L-Y, Ren Z-F: **Night-shift work, sleep duration, daytime napping, and breast cancer risk**. *Sleep Medicine* 2015, **16**(4):462-468.
5. Yang W, Shi Y, Ke X, Sun H, Guo J, Wang X: **Long-term sleep habits and the risk of breast cancer among Chinese women: a case–control study**. *European Journal of Cancer Prevention* 2019, **28**(4).
6. Akerstedt T, Knutsson A, Narusyte J, Svedberg P, Kecklund G, Alexanderson K: **Night work and breast cancer in women: a Swedish cohort study**. *BMJ Open* 2015, **5**(4):e008127-e008127.
7. Jones ME, Schoemaker MJ, McFadden EC, Wright LB, Johns LE, Swerdlow AJ: **Night shift work and risk of breast cancer in women: the Generations Study cohort**. *British Journal of Cancer* 2019, **121**(2):172-179.
8. Koppes LLJ, Geuskens GA, Pronk A, Vermeulen RCH, De Vroome EMM: **Night work and breast cancer risk in a general population prospective cohort study in The Netherlands**. *European Journal of Epidemiology* 2014, **29**(8):577-584.
9. McNeil J, Heer E, Willemsen RF, Friedenreich CM, Brenner DR: **The effects of shift work and sleep duration on cancer incidence in Alberta`s Tomorrow Project cohort**. *Cancer Epidemiology* 2020, **67**:101729.

10. Pronk A, Ji BT, Shu XO, Xue S, Yang G, Li HL, Rothman N, Gao YT, Zheng W, Chow WH: **Night-Shift Work and Breast Cancer Risk in a Cohort of Chinese Women**. *American Journal of Epidemiology* 2010, **171**(9):953-959.

11. Sweeney MR, Sandler DP, Niehoff NM, White AJ: **Shift Work and Working at Night in Relation to Breast Cancer Incidence**. *Cancer Epidemiology Biomarkers & Prevention* 2020, **29**(3):687-689.

12. Travis RC, Balkwill A, Fensom GK, Appleby PN, Reeves GK, Wang X-S, Roddam AW, Gathani T, Peto R, Green J *et al*: **Night Shift Work and Breast Cancer Incidence: Three Prospective Studies and Meta-analysis of Published Studies**. *Journal of the National Cancer Institute* 2016, **108**(12):djw169.

13. Harma M, Ojajarvi A, Koskinen A, Lie JA, Hansen J: **Shift work with and without night shifts and breast cancer risk in a cohort study from Finland**. *Occupational and Environmental Medicine* 2022.

14. Schernhammer ES, Kroenke CH, Laden F, Hankinson SE: **Night work and risk of breast cancer**. *Epidemiology* 2006, **17**(1):108-111.

15. Schernhammer ES, Laden F, Speizer FE, Willett WC, Hunter DJ, Kawachi I, Colditz GA: **Rotating night shifts and risk of breast cancer in women participating in the nurses' health study**. *J Natl Cancer Inst* 2001, **93**(20):1563-1568.

16. Davis S, Mirick DK, Stevens RG: **Night Shift Work, Light at Night, and Risk of Breast Cancer**. *JNCI: Journal of the National Cancer Institute* 2001, **93**(20):1557-1562.

17. Fritschi L, Erren TC, Glass DC, Girschik J, Thomson AK, Saunders C, Boyle T, El-Zaemey S, Rogers P, Peters S *et al*: **The association between different night shiftwork factors and breast cancer: a case–control study**. *British Journal of Cancer* 2013, **109**(9):2472-2480.

18. Grundy A, Richardson H, Burstyn I, Lohrisch C, SenGupta SK, Lai AS, Lee D, Spinelli JJ, Aronson KJ: **Increased risk of breast cancer associated with long-term shift work in Canada**. *Occupational and Environmental Medicine* 2013, **70**(12):831.

19. Menegaux F, Truong T, Anger A, Cordina-Duverger E, Lamkarkach F, Arveux P, Kerbrat P,

Févotte J, Guénel P: **Night work and breast cancer: A population-based case-control study in France (the CECILE study)**. *International Journal of Cancer* 2013, **132**(4):924-931.

20. O'Leary ES, Schoenfeld ER, Stevens RG, Kabat GC, Henderson K, Grimson R, Gammon MD, Leske MC, Fields ftE, Group BCoLIS: **Shift Work, Light at Night, and Breast Cancer on Long Island, New York**. *American Journal of Epidemiology* 2006, **164**(4):358-366.

21. Papantoniou K, Castaño-Vinyals G, Espinosa A, Aragonés N, Pérez-Gómez B, Ardanaz E, Altzibar JM, Sanchez VM, Gómez-Acebo I, Llorca J *et al*: **Breast cancer risk and night shift work in a case–control study in a Spanish population**. *European Journal of Epidemiology* 2016, **31**(9):867-878.

22. Pesch B, Harth V, Rabstein S, Baisch C, Schiffermann M, Pallapies D, Bonberg N, Heinze E, Spickenheuer A, Justenhoven C *et al*: **Night work and breast cancer – results from the German GENICA study**. *Scandinavian Journal of Work, Environment & Health* 2010(2):134-141.

23. Pham T-T, Hwang M, Lee E-S, Kong S-Y, Jung S-Y, Lee S, Kim J, Ha M, Kim S-Y, Park B: **Night-shift work and risk of breast cancer in Korean women**. *Clinical Epidemiology* 2019, **Volume 11**:743-751.

24. Hansen J, Lassen CF: **Nested case–control study of night shift work and breast cancer risk among women in the Danish military**. *Occupational and Environmental Medicine* 2012, **69**(8):551-556.

25. Lie J-AS, Roessink J, Kjærheim K: **Breast Cancer and Night Work among Norwegian Nurses**. *Cancer Causes & Control* 2006, **17**(1):39-44.

26. Szkiela M, Kusidel E, Makowiec-Dabrowska T, Kaleta D: **How the intensity of night shift work affects breast cancer risk**. *International Journal of Environmental Research and Public Health* 2021, **18**(9):4570.

27. Hansen J, Stevens RG: **Case-control study of shift-work and breast cancer risk in Danish nurses: Impact of shift systems**. *European Journal of Cancer* 2012, **48**(11):1722-1729.

28. Haus EL, Smolensky MH: **Shift work and cancer risk: potential mechanistic roles of

circadian disruption, light at night, and sleep deprivation. *Sleep Med Rev* 2013, **17**(4):273-284.

29. National_Toxicology_Program. In: *NTP Cancer Hazard Assessment Report on Night Shift Work and Light at Night.* edn. Research Triangle Park (NC): National Toxicology Program; 2021.

30. Urbano T, Vinceti M, Wise LA, Filippini T: **Light at night and risk of breast cancer: a systematic review and dose-response meta-analysis**. *Int J Health Geogr* 2021, **20**(1):44.

31. Flynn-Evans EE, Stevens RG, Tabandeh H, Schernhammer ES, Lockley SW: **Total visual blindness is protective against breast cancer**. *Cancer Causes & Control* 2009, **20**(9):1753-1756.

32. Pukkala E, Ojamo M, Rudanko S-L, Stevens RG, Verkasalo PK: **Does Incidence of Breast Cancer and Prostate Cancer Decrease with Increasing Degree of Visual Impairment**. *Cancer Causes & Control* 2006, **17**(4):573-576.

33. Kliukiene J, Tynes T, Andersen A: **Risk of breast cancer among Norwegian women with visual impairment**. *British Journal of Cancer* 2001, **84**(3):397-399.

34. Verkasalo PK, Pukkala E, Stevens RG, Ojamo M, Rudanko SL: **Inverse association between breast cancer incidence and degree of visual impairment in Finland**. *British Journal of Cancer* 1999, **80**(9):1459-1460.

35. Feychting M, OsterLund B, Ahlbom A: **Reduced Cancer Incidence among the Blind**. *Epidemiology* 1998, **9**(5):490-494.

36. Hong J, He Y, Fu R, Si Y, Xu B, Xu J, Li X, Mao F: **The relationship between night shift work and breast cancer incidence: A systematic review and meta-analysis of observational studies**. *Open Med (Wars)* 2022, **17**(1):712-731.

37. Rabstein S, Harth V, Pesch B, Pallapies D, Lotz A, Justenhoven C, Baisch C, Schiffermann M, Haas S, Fischer HP *et al*: **Night work and breast cancer estrogen receptor status--results from the German GENICA study**. *Scand J Work Environ Health* 2013, **39**(5):448-455.
